# Repertoire of BALB/c Mice Natural Anti-Carbohydrate Antibodies: Mice vs. Humans Difference, and Otherness of Individual Animals

**DOI:** 10.3389/fimmu.2017.01449

**Published:** 2017-11-06

**Authors:** Daniel Bello-Gil, Nailya Khasbiullina, Nadezhda Shilova, Nicolai Bovin, Rafael Mañez

**Affiliations:** ^1^Infectious Pathology and Transplantation Division, Bellvitge Biomedical Research Institute (IDIBELL), Hospitalet de Llobregat, Spain; ^2^Shemyakin-Ovchinnikov Institute of Bioorganic Chemistry, Russian Academy of Sciences, Moscow, Russia; ^3^Intensive Care Department, Bellvitge University Hospital, Hospitalet de Llobregat, Spain

**Keywords:** printed glycan array technology, glycochips, BALB/c, humans, natural antibodies repertoire

## Abstract

One of the most common genetic backgrounds for mice used as a model to investigate human diseases is the inbred BALB/c strain. This work is aimed to characterize the pattern of natural anti-carbohydrate antibodies present in the serum of 20 BALB/c mice by printed glycan array technology and to compare their binding specificities with that of human natural anti-carbohydrate antibodies. Natural antibodies (NAbs) from the serum of BALB/c mice interacted with 71 glycans from a library of 419 different carbohydrate structures. However, only seven of these glycans were recognized by the serum of all the animals studied, and other five glycans by at least 80% of mice. The pattern of the 12 glycans mostly recognized by the circulating antibodies of BALB/c mice differed significantly from that observed with natural anti-carbohydrate antibodies in humans. This lack of identical repertoires of natural anti-carbohydrate antibodies between individual inbred mice, and between mice and humans, should be taken into consideration when mouse models are intended to be used for investigation of NAbs in biomedical research.

## Introduction

Antibody repertoire has marked the success and perpetuity of species. There is a group of circulating antibodies known as natural antibodies (NAbs) present in blood at early life without any previous immunogenic challenge (1, 2). NAbs are spontaneously produced primarily by B-1 cells and their levels, and antigen affinities, remain almost constant during lifetime (3). NAbs (mostly IgM) are encoded by their genes in germline configuration by B cells, which have not been subjected to somatic hypermutation and affinity maturation (4). In fact, at least 80% of the serum IgM, in healthy conditions, is produced by this way (5). Little is known about factors involved in the regulation of composition of circulating NAbs. Its origin, repertoire, and physiological role are still controversial and an issue of continued debate (6).

The most expanded origin hypothesis suggests that stimulation of B-1 lymphocytes is produced by exposition to microbiota antigens (7). NAbs were highlighted by the discovery in the early twentieth century of the ABO antigen system in human blood. After that, a large group of NAbs has been described in humans, which include other alloantibodies related to blood group antigens (Rh, Lewis, etc.), xenoantibodies, and antibodies that target tumor-associated antigens (3, 8). In general, NAbs show a polyreactive binding as they react to similar epitopes on a variety of molecular entities (9, 10). The maintenance of immune homeostasis through the defense against foreign invaders and own damaged/apoptotic cells, and the housekeeping removal of cellular debris or metabolite clearance, are functions attributed to NAbs (10, 11). Most of these antibodies target carbohydrate structures, and have been reported to play protective, but also pathogenic roles, in both autoimmune and inflammatory diseases (12, 13). Therefore, an understanding of the composition and function of the glycan-reactive NAb repertoire in a healthy condition continues being an issue of paramount importance (13).

The Printed Glycan Array (PGA) technology has a high sensitivity and offers the possibility to analyze hundreds of different glycan antigens to explore circulating natural anti-carbohydrate antibodies in different species (8, 9, 14, 15). This allows the minimization of one of the major problems associated with the analysis of anti-carbohydrate antibodies; the cross-reactivity of a particular antibody with different glycans (16). Mice and specifically the BALB/c strain is one of the animal species more often used as a model of human diseases in both cancer and immunology research (17). Although there are previous reports regarding global analysis of the natural antibody repertoire (18–20), little is known about the exact specificities targeted by natural anti-carbohydrate antibodies in these animals, and which of them are shared with humans. The study presented by Dai et al. (20) is limited to a reduced number of glycan moieties, including four representative carbohydrate structures: homo-polysaccharides of 1,4-linked-d-galactopyranosyluronic acids, 1,6-glucan (dextran), 1,3-mannan and β-glucan. From these glycan structures, mannan was not recognized by serum Abs from any of the mouse and rat strains examined and some variability regarding of glycan recognition among mice strains under examination was reported. Despite this, the authors concluded that IgM reactivity repertoires against glycan antigens in rodents are practically homogeneous within inbred strains and largely conserved in the species.

The present work is aimed to describe the natural anti-carbohydrate antibody repertoire of BALB/c mice by PGA, using a library of 419 different fully characterized glycan structures, and to compare their binding specificities with that of human natural anti-carbohydrate antibodies.

## Materials and Methods

### Ethics Statement

Animals were handled in strict accordance with good animal practice as defined by the relevant local animal welfare bodies. All animal procedures were supervised and approved by Bellvitge Biomedical Research Institute (IDIBELL) ethics committee for animal experimentation and the Catalonia Government. The care and handling of the animals were conformed to the Guide for the Care and Use of Laboratory Animals published by the US National Institutes of Health (NIH Publication no. 85-23 revised 1996) and the European Agreement on Vertebrate Animal Protection for Experimental Use (86/609).

### BALB/c Mice

BALB/c mice 10-week-old (Harlan, France), 13 female and 7 male, were maintained in separated cages at IDIBELL animal facility (specific pathogen free, SPF) under controlled conditions of temperature (21 ± 1°C), humidity (55 ± 5%), cycles of light/dark of 12/12 h, and with food and water given *ad libitum*.

### Serum Collection and Processing

Mice blood extraction was made without the need of anesthesia by submandibular bleeding (21). Serum was collected by mild centrifugation (10 min, 1,200 *g* at 4°C) and stored at −80°C for further analysis. The human serum was collected from 11 human healthy donors, processed and stored under similar condition by Semiotik LLC.

### Glycan Array Analysis

Glycochips were prepared by Semiotik LLC (Moscow, Russia) from 419 different synthetic amine-functionalized glycans, using *N*-hydroxysuccinimide-derivatized glass slides (slide H, Schott-Nexterion, Mainz, Germany), as described in Ref. (3, 15). The glycan library included blood group antigens and some of the most frequently occurring terminal oligosaccharides, as well as core motifs of mammalian *N*- and *O*-linked glycoproteins and glycolipids, tumor-associated carbohydrate antigens, and polysaccharides from pathogenic bacteria. Synthetic glycan structures (>95% purity) are structurally the same as natural ones. Structures and NMR data of polysaccharides and related references are in http://csdb.glycoscience.ru/bacterial (Zelinsky Institute of Organic chemistry, Moscow, Russia). All glycans were printed in six replicates. After printing, glycochips were incubated in an incubation chamber for 15 min at 25°C with PBS plus 0.1% (v/v) Tween-20 (buffer 3) (Sigma-Aldrich, St. Louis, MO, USA), and the buffer was then carefully removed from the microchip surface using Whatman^®^ filter paper (Sigma-Aldrich, St. Louis, MO, USA). BALB/c mouse sera were diluted (1:15) in PBS plus 1% (w/v) bovine serum albumin (BSA; Sigma-Aldrich, St. Louis, MO, USA) and 1% (v/v) Tween-20 (buffer 1). Diluted serum was spread over the slide surface and incubated with agitation (30 rpm) at 37°C for 1.5 h. After a round of washing steps with buffer 3, buffer 4 (PBS with 0.001% v/v Tween-20), and distilled water (Milli-Q grade), the glycochips were drained by mild centrifugation (1 min, 175 *g*, Eppendorf, Hamburg, Germany). The glycochips were then incubated for 1 h at 37°C (30 rpm) with goat anti-mouse IgG + IgM (H + L) conjugated to biotin (Thermo Fisher Scientific, Waltham, MA, USA) and diluted 200-fold in PBS plus 1% BSA and 0.1% Tween-20 (buffer 2). The unbound fraction was removed by repeating the same round of washing steps. Glass slides were incubated in darkness at 25°C for 45 min (30 rpm) with streptavidin labeled with Cy5 dye (GE Healthcare, Little Chalfont, Buckinghamshire, UK) and diluted 1:500 in buffer 2. After another round of washing, the glycochips were dried by airflow in darkness. Finally, the glycochips were scanned using a ScanArray GX Plus scanner (PerkinElmer, Waltham, MA, USA) with a laser (excitation wavelength of 633 nm). All data analysis was performed with the ScanArray^®^ Express Microarray Analysis System (PerkinElmer, Waltham, MA, USA). The binding results were expressed in relative fluorescence units (RFU) as median ± median absolute deviation (MAD). Interactive exploration of multidimensional data (heat mapping and clustering analysis) was performed with the Hierarchical Clustering Explorer application developed by the University of Maryland, MD, USA.^1^

## Results

To avoid potentially confounding differences in genetic backgrounds, BALB/c mice were taken from inbred SPF populations (Harlan, France). The repertoire of circulating natural anti-carbohydrate antibodies was studied by PGA technology using a library of 419 different glycan structures. Although a previous study (22) showed that in normal mouse sera IgM can bind the F(ab′)_2_ of natural IgG autoantibodies, thus the dilution of normal serum decreases such IgM anti-IgG autoantibodies and unmasks these natural IgG autoantibodies, we have demonstrated that this dilution dependent effect is not present in our test system (15). Hence, we have used diluted sera (1:15) in all PGA determinations. In the case of mice due to the constraint in the serum amount, IgG and IgM anti-carbohydrate antibodies were simultaneously determined. Structural identity of polysaccharides was confirmed by NMR (deposited in http://csdb.glycoscience.ru/bacterial). All carbohydrates used in the PGA structurally were the same as natural ones. However, density in the slide, length of spacer or type of carrier (protein or another polymer, peptide, etc.) can influence their activity. Therefore, their presentation on the array is quite possibly far from natural presentation (23, 24). Nevertheless, this constraint of our *in vitro* approach had no impact on the main objective of our work because both sets of PGA determinations (humans and mice) were carried out using the same glycan library and conditions; hence, were similarly affected.

The data resulting from PGA experiments have been deposited in *NCBI GEO Database* (25) with the name “Repertoire of BALB/c mice natural anti-carbohydrate antibodies,” and are accessible through GEO Series accession number GSE97151.^2^

**Figure 1:**
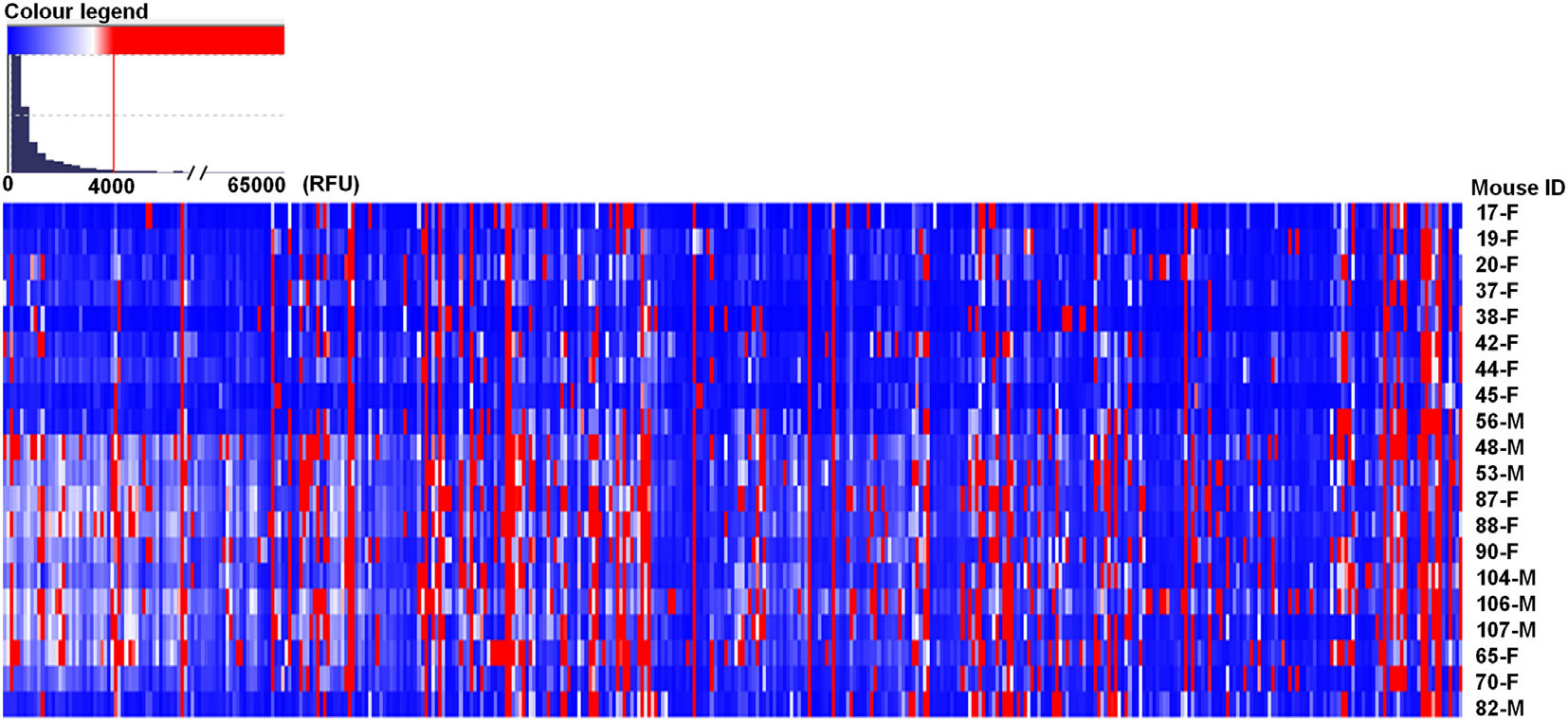
BALB/c mice showed different repertoire of natural circulating anti-carbohydrate antibodies. Mouse (*n* = 20) serum (1:15) was incubated with chips printed with 419 glycans. Chips were scanned using a ScanArray GX Plus reader and data were analyzed with the ScanArray^®^ Express Microarray Analysis System (PerkinElmer). The binding results for IgM + IgG were expressed in relative fluorescence units (RFU) as median ± median absolute deviation (MAD). In the heat map, blue and white colors represent binding signals, in RFU, lower than 4,000 (background); red color signals ≥4,000 RFU (positive binding). F, female; M, male.

In the PGA, we considered values above 4,000 RFU as a positive signal of antibody binding (this value is ~10% of the top glycans RFU), which were expressed as the median ± median absolute deviation (MAD) (Table S1 in Supplementary Material). The majority of printed glycans were not targeted by any natural antibody present in the serum of BALB/c mice (Figure 1, in blue), and 71 carbohydrates (Figure 1, in red) demonstrated ≥4,000 RFU in the PGA (see NCBI GEO Database: GSE97151). The top rank glycans included 12 with median signal intensities of bound antibodies ≥10,000 RFU (Table 1). Seven of them were recognized by serum antibodies from all the mice involved in the study, while other five glycans were targeted by serum samples of at least 80% of animals (Table 1). Sulfated glycans comprised 50% of the high-binding glycans and βGal-terminated oligosaccharides 25%. About gender, we did not observe marked differences between male and female in the majority of top rank glycans listed in Table 1.

**Table 1:** Specificity of carbohydrates targeting natural antibodies in BALB/c mice.

Glycan ID (#)	Structure	Common name	Median and MAD as RFU		Number of mice showing RFU ≥4,000 (%)	Number of human donors showing RFU ≥4,000 (%)	
						IgM	IgG
060	6-O-Su-Galβ-sp^a^		61,113	1,156	100	73	0
271	Galβ1-6Galβ1-4Glcβ-sp		53,622	1,934	100	55	27
802	Galβ1-3GalNAc(fur)β-sp		51,348	2,324	100	73	9
176	3-O-Su-Galβ1-4(6-O-Su)Glcβ-sp		43,008	9,342	100	9	0
166	GlcAβ1-6Galβ-sp		39,105	2,993	85	18	0
150	3-O-Su-Galβ1-3GalNAcα-sp		37,943	3,232	100	18	0
437	GalNAcα1-3(Fucα1-2)Galβ1-3GalNAcβ-sp	A(type 4)	33,886	3,193	90	45	45
125	6-Bn-Galβ1-4GlcNAcβ-sp		32,674	5,389	95	0	0
154	3-O-Su-Galβ1-3GlcNAcβ-sp		32,651	3,954	100	64	36
177	3-O-Su-Galβ1-4(6-O-Su)GlcNAcβ-sp		32,496	7,215	100	9	9
287	3-O-Su-Galβ1-3(Fucα1-4)GlcNAcβ-sp	SuLe^a^	20,063	4,962	95	0	9
234	Galβ1-4(Fucα1-3)GlcNAcβ-sp	Le^x^	13,573	2,635	80	0	0

*List of glycans with binding signals above 4,000 relative fluorescence units (RFU) in at least 80% of examined mice (*n* = 20). Comparison with human data (*n* = 11). The data obtained by Semiotik LLC, Russia, in the same conditions*.

*^a^sp means aminoethyl, aminoprolyl, or glycyl spacer*.

**Figure 2:**
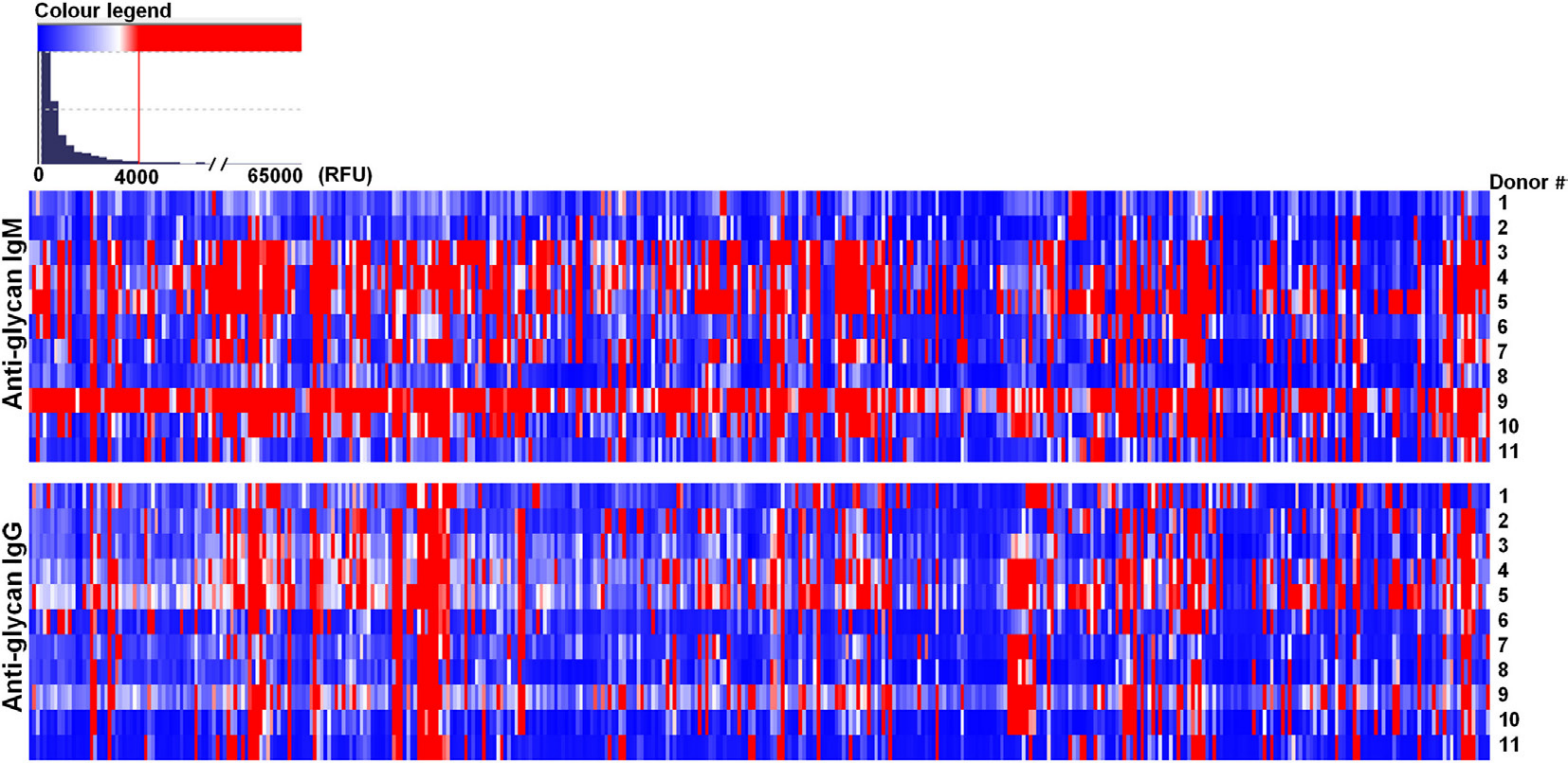
Humans showed different repertoire of natural circulating anti-carbohydrate antibodies. Human (*n* = 11) serum (1:15) was incubated with chips printed with 419 glycans. Chips were scanned using a ScanArray GX Plus reader and data were analyzed with the ScanArray^®^ Express Microarray Analysis System (PerkinElmer). The binding results for IgM and IgG were expressed in relative fluorescence units (RFU) as median ± median absolute deviation (MAD). In the heat map blue and white colors represent binding signals, in RFU, lower than 4,000 (background); red color signals ≥4,000 RFU (positive binding).

Most of the circulating anti-glycan antibodies found in mice were not widely represented in the human sera, as the number of human donors showing signals ≥4,000 RFU in the PGA was limited or absent for the major part of these top rank glycan structures (Table 1). Concomitantly, the high level circulating anti-glycan antibodies found in humans (Table 2) were poorly represented among the animals assessed. Additionally, humans showed, like mice, significant variability between individuals in the level and diversity of circulating anti-glycan antibodies (Figure 2; Table S2 in Supplementary Material).

**Table 2:** Specificity of carbohydrates targeting natural antibodies in humans.

Glycan ID (#)	Structure	Common name	Number of human donors showing RFU ≥4,000 (%)		Number of mice showing RFU ≥4,000 (%)
			IgM	IgG	
019	ManNAcβ-sp^a^		91	91	20
080	Galα1-3GlcNAcβ-sp		82	82	0
082	Galα1-4GlcNAcβ-sp	αLN	73	73	5
101	GalNAcα1-3GalNAcβ-sp	Fs-2	82	91	5
149	GlcNAcβ1-4(6-O-Su)GlcNAcβ-sp		82	82	25
246	GlcNAcβ1-2Galβ1-3GalNAcα-sp		91	82	0
256	GlcNAcβ1-6(GlcNAcβ1-4)GalNAcα-sp		91	91	40
278	GalNAcα1-3GalNAcβ1-3Galβ-sp	Fs-3	73	82	n.d
375	Galα1-4GlcNAcβ1-3Galβ1-4GlcNAcβ-sp		73	73	5
378	Galβ1-3GlcNAcα1-3Galβ1-4GlcNAcβ-sp		82	73	45
399	Galβ1-3GlcNAcα1-3Galβ1-3GlcNAcβ-sp		82	82	50
806	Galα1-6Glcα-sp		82	73	20
808	Galα1-6Glcβ-sp	Melibiose	91	73	35

*List of glycans with binding signals above 4,000 relative fluorescence units (RFU) for both, IgM and IgG antibodies in at least 70% of human donors (*n* = 11). Comparison with mice data (*n* = 20). The data obtained by Semiotik LLC, Russia, in the same conditions*.

*^a^sp means aminoethyl, aminoprolyl, or glycyl spacer*.

*n.d, not determined*.

## Discussion

Murine and specifically BALB/c mice are among the most widely used inbred strains for animal experimentation to address almost every aspect of human health (17). This work demonstrates that genetically identical SPF mice should not be considered as “totally equivalents” from the immunological view as they present, despite some conservatism, different patterns of natural circulating anti-carbohydrate antibodies, which also differ dramatically from the conserved anti-carbohydrate antibody repertoire found in humans. Previous global analysis of natural antibody repertoires has revealed a marked conservation of reactivity patterns within inbred mouse strains (18–20). However, some of these studies must be analyzed with caution due to the limited number of glycans assessed (20). This homogeneity among genetically identical animals was not observed in our study (25), and could be explained by the differences demonstrated in the analysis of the gut microbial population of inbred animals (26). If the production of natural anti-carbohydrate antibodies is triggered by the antigenic stimulation of microbiota, and this is different among inbred mice, fine specificity of these antibodies will not be identical.

There are also significant differences between mice and humans regarding the primary glycan specificities targeted by natural anti-carbohydrate antibodies. The discrepancy in repertoires is quite evident if top rank carbohydrates recognized by mice antibodies are directly compared with the top rank of circulating anti-glycan antibodies in humans (3). This disparity cannot be attributed to alloantibodies because in humans anti-blood group antibodies (like anti-A, anti-B, anti-Lewis) are not top rank immunoglobulins (3). The most intriguing appear to be the rather high level of anti-Le^X^ in BALB/c mice. In humans, healthy donors never have antibodies to Le^X^ epitope, Galβ1-4(Fucα1-3)GlcNAcβ, as well as to related so-called type 2 motif containing antigens (i.e., structures with Galβ1-4GlcNAc core) like Le^Y^ and SiaLe^X^. The absence of these antibodies in humans is easy to explain: Le^X^ termination is known as the structure of many glycoproteins and glycolipids of endothelial and blood group cells membrane. In contrast to humans, 80% of BALB/c mice demonstrated moderate levels of anti-Le^X^ antibodies. The “moderate” means a level comparable or higher than, for example, titers of anti-A/B alloantibodies, or anti-αGal xenoantibodies, which cause hemolytic reactions or organ rejection in humans. In mice, Le^X^ is known as stage-specific embryonic antigen-III and plays a crucial role in neurogenesis, embryogenesis, and reproduction system (27, 28). Why in BALB/c mice the antibodies coexist with cognate antigen without immunologic attack remains unclear. Notably, other mice strains (29) also found to have anti-Le^X^ NAbs, with an apparent function to protect from Schistosoma parasites (30). Concurrently, top anti-glycan antibodies conserved among humans (3, 8), such as GlcNAcβ-terminated, GlcNAcα-terminated, Rha, Le^C^, Fs/A_di_, asialo-GM1, and Fucα1-3(4)GlcNAc, are completely missing in mice, or, like in case of anti-blood group P_1_ and P^k^ trisaccharides, at very low level. Although we show with the glycan array that BALB/c and human IgM have different glycan binding specificities, these differences may not significantly alter the functional effect of human or mouse IgM on murine cells as shown in prior studies (31–33).

The differences observed in the repertoire of anti-carbohydrate antibodies in genetically identical BALB/c mice, and between these mice and humans, also reflect the uncertainties about the functional role and origin of NAbs. Three hypotheses attempt to explain the development of these antibodies (6). The first suggests the specific stimulation by new antigens of the bacterial microbiota; the second is based on the response to endogenous degradation products of normal cells, not to neoantigens; and the third proposes that NAbs result from the exposure to molecular patterns. The latter are different conserved molecules located close to each other that can be divided into two groups: MAMPs, microorganism associated molecular patterns, composed of polysaccharides, and DAMPs, damage associated molecular patterns, constituted by proteins. In the case of polysaccharides, human anti-glycan antibodies, including anti-A/B allo-agglutinins, antibodies to glycoprotein *O*-chain glycans Galβ1-3GalNAcα (TF) and GalNAcα (Tn), are risen due to contact of the newborn immune system with intestinal microbiota (34, 35). Pivotal role in this phenomenon might play bacterial polysaccharides, structure of which mimics ABH blood groups (7, 36), TF/Tn or other related mammalian glycans (37, 38). From the moment of birth, the gastrointestinal tract and respiratory system in mammals are actively colonized by bacteria. About 103 species of non-pathogenic (commensal) bacteria form the basis of normal intestinal microbiota (39), although the total number of species is estimated to be greater. These bacteria possess millions of antigens, and they are capable to prime those B-1 lymphocytes which are genetically selected for the synthesis of NAbs (6, 40). It was shown that up to 90% of the immunoglobulin-secreting cells of the normal mouse intestine produce natural Abs that are absent in germ-free mice (41, 42). Thus, the appearance of a particular natural anti-carbohydrate antibody requires “two keys”—the existence of B-1 cell gene and the priming with bacterial antigen (a mimotope of the cognate antigen). Bacteria are the best source for anti-carbohydrate antibody priming for two additional reasons: (1) appearance only after birth, (2) the need of toll-like receptors for recognition by B-1 cells; this mechanism excludes priming of B-1 cells with auto-antigens at the embryonic stage (6). Genetics of B-cells, as well as microbiotas of humans and mice are different, so it is not surprising that the resulting repertoires of NAbs are not similar. At the same time, since some of the NAbs (for instance, anti-A/B) play a similar physiologically active role, they are similar in different species.

In summary, the results presented here indicate that the repertoires of circulating natural anti-glycan antibodies in BALB/c mice appear to be not identical for genetically identical individual animals. Additionally, mice antibody repertoire shows significant differences to that present in humans, suggesting a caution when using mice as an animal model for investigation of human NAbs for biomedical studies.

## Ethics Statement

Animals were handled in strict accordance with good animal practice as defined by the relevant local animal welfare bodies. All animal procedures were supervised and approved by Bellvitge Biomedical Research Institute (IDIBELL) ethics committee for animal experimentation and the Catalonia Government. The care and handling of the animals were conformed to the Guide for the Care and Use of Laboratory Animals published by the US National Institutes of Health (NIH Publication no. 85-23 revised 1996) and the European Agreement of Vertebrate Animal Protection for Experimental Use (86/609).

## Author Contributions

Contributions of the authors can be summarized as follows: performed the experiments: DB-G and NK. Analyzed the data: DB-G, NK, NS, NB, and RM. Contributed reagents/materials/analysis tools: NB and RM. Wrote the article: DB-G. Contributed with ideas: DB-G, NK, NS, NB, and RM.

## Conflict of Interest Statement

The authors declare that the research was conducted in the absence of any commercial or financial relationships that could be construed as a potential conflict of interest.

## Footnotes

^1^http://www.cs.umd.edu/hcil/hce/.

^2^https://www.ncbi.nlm.nih.gov/geo/query/acc.cgi?acc=GSE97151.
