# Supplementary material for: Repertoire of BALB/c Mice Natural Anti-Carbohydrate Antibodies: Mice vs. Humans Difference, and Otherness of Individual Animals
Source: Front Immunol. 2017 Nov 6;8:1449. doi: 10.3389/fimmu.2017.01449 (PMC5681490; doi:10.3389/fimmu.2017.01449)
Supplement: Supplementary file 1 [file data_sheet_1.pdf]

Supplementary Material

Repertoire of BALB/c mice natural anti-carbohydrate antibodies: mice vs. humans difference, and otherness of individual animals

Daniel Bello-Gil[1,*], Nailya Khasbiullina[2], Nadezhda Shilova[2], Nicolai Bovin[2] and Rafael Mañez[1,3]

*** Correspondence:** dbello@idibell.cat

Table S1. List of glycans, their binding to natural circulating antibodies (IgM+IgG) of BALB/c mice (n=20), expressed in relative fluorescence units (RFU) as median ± median absolute deviation (MAD), and the number of animals exceeding *cut off* (≥ 4000 RFU).

Glycan ID (#)	Structure	Common Name	Median and MAD as RFU		Animals showing RFU ≥ 4000
001	Fucα-sp3		1590	342	1
002	Galα-sp3		1522	316	0
003	Galβ-sp3		11858	1420	12
004	GalNAcα1-OSer	T_nSer	2145	395	2
005	GalNAcα-sp3	T_n	1642	192	2
006	GalNAcβ-sp3		1001	250	0
007	Glcα-sp3		1242	222	0
009	Glcβ-sp3		1576	325	0
010	GlcNAcβ-sp3		4124	471	1
011	GlcNAcβ-sp2		4634	607	2
012	GlcNAcβ-sp7		2068	523	2
013	GlcNAcβ-sp8		7267	1117	9
014	GlcN(Gc)β-sp4		914	203	0
015	$HOCH_2(HOCH)_4CH_2NH_2$	aminoglucitol	1374	316	0
016	Manα-sp3		1164	263	0
017	Manα-sp4		1567	369	0
018	Manβ-sp4		1842	319	2
019	ManNAcβ-sp4		4752	390	4
020	Rhaα-sp3		970	308	0

021	Galβ-sp4		1338	269	0
022	GlcNAcβ-sp4		932	230	0
023	GalNAcβ-sp4		1246	271	0
024	GlcNAcα-sp3		1463	260	2
025	GalNAcβ-sp10		848	209	0
026	Rhaβ-sp4		1098	227	0
027	3,6-Me2Glc	DMG	673	123	0
028	Xylβ-sp4		1411	302	0
029	Fucβ-sp4		1139	301	0
030	Glcβ-sp4		1449	429	1
031	L-Araα-sp4		1099	369	0
032	GalNGcβ-sp3		483	119	0
037	3-O-Su-Galβ-sp3		2317	503	3
038	3-O-Su-GalNAcβ-sp3		3657	689	7
041	6-O-Su-GalNAcα-sp3		5504	1443	7
043	6-O-Su-GlcNAcβ-sp3		1040	253	1
044	GlcAα-sp3	α-glucuronic acid	1275	327	0
045	GlcAβ-sp3	β-glucuronic acid	1671	335	1
046	6-H_2PO_3Glcβ-sp4	β-Glc6P	1369	316	0
047	6-H_2PO_3Manα-sp3	α-Man6P	1300	322	0
048	Neu5Acα-sp3		583	131	0
049	Neu5Acα-sp9		1349	334	3
050	Neu5Acβ-sp3		2184	526	3
051	Neu5Acβ-sp9		1698	609	3
052	Neu5Gcα-sp3		847	286	0
053	Neu5Gcβ-sp3		1202	307	0
054	9-NAc-Neu5Acα-sp3		256	77	0
055	3-O-Su-GlcNAcβ-sp3		840	214	0
056	Galα-sp7		1016	263	0
057	Rib -sp4		1153	248	0
058	Fucβ-sp3		2266	422	1
059	Crypted	Crypted	1360	389	0
060	6-O-Su-Galβ-sp3		61113	1156	20
061	3-OSu-GalNAcα-sp3		2692	622	3
071	Fucα1-2Galβ-sp3	H_{di}	1077	245	1
072	Fucα1-3GlcNAcβ-sp3		1424	300	2

073	Fucα1-4GlcNAcβ-sp3	Le	755	217	0
074	Fucβ1-3GlcNAcβ-sp3		533	130	0
075	Galα1-2Galβ-sp3		790	184	0
076	Galα1-3Galβ-sp3	B_{di}	775	192	0
077	Galα1-3GalNAcβ-sp3	$T_{\alpha\beta}$	668	155	0
078	Galα1-3GalNAcα-sp3	$T_{\alpha\alpha}$	581	113	0
080	Galα1-3GlcNAcβ-sp3		384	76	0
081	Galα1-4GlcNAcβ-sp3	α-LN	815	183	1
082	Galα1-4GlcNAcβ-sp8	α-LN	713	144	1
083	Galα1-6Glcβ-sp4	melibiose	2285	348	2
084	Galβ1-2Galβ-sp3		1332	301	1
086	Galβ1-3GlcNAcβ-sp2	Le^{C}	609	130	0
087	Galβ1-3Galβ-sp3		1488	282	1
088	Galβ1-3GalNAcβ-sp3	$T_{\beta\beta}$	1468	174	2
089	Galβ1-3GalNAcα-sp3	TF	297	59	0
092	Galβ1-4Glcβ-sp2	Lactose, Lac	889	182	0
093	Galβ1-4Glcβ-sp4	Lactose, Lac	1089	290	0
094	Galβ1-4Galβ-sp4		1124	278	0
096	Galβ1-4GlcNAcβ-sp2	N-acetyllactosamine, LN	2604	893	1
097	Galβ1-4GlcNAcβ-sp3	N-acetyllactosamine, LN	491	103	0
098	Galβ1-4GlcNAcβ–sp5	N-acetyllactosamine, LN	341	65	0
099	Galβ1-4GlcNAcβ-sp8	N-acetyllactosamine, LN	357	65	1
100	Galβ1-6Galβ-sp4		18406	2396	15
101	GalNAcα1-3GalNAcβ-sp3	Fs-2	1158	307	1
102	GalNAcα1-3Galβ-sp3	A_{di}	1199	295	1
103	GalNAcα1-3GalNAcα-sp3	core 5	451	87	0
104	GalNAcβ1-3Galβ-sp3		603	144	0
105	GalNAcβ1-3GalNAcβ-sp3	para-Fs	5975	1065	11
106	GalNAcβ1-4GlcNAcβ-sp3	LacdiNAc	332	79	0
107	GalNAcβ1-4GlcNAcβ-sp2	LacdiNAc	390	95	0
110	Glcα1-4Glcβ-sp3	maltose	10870	2172	10
111	Glcβ1-4Glcβ-sp4	cellobiose	3363	407	3
112	Glcβ1-6Glcβ-sp4	gentiobiose	6968	1229	5
113	GlcNAcβ1-3GalNAcα-sp3	core 3	4852	620	3
114	GlcNAcβ1-3Manβ-sp4		1474	423	2
115	GlcNAcβ1-4GlcNAcβ-Asn	chitobiose-Asn	12575	2669	14
116	GlcNAcβ1-4GlcNAcβ-sp3	chitobiose	1040	186	1

117	GlcNAcβ1-4GlcNAcβ-sp4	chitobiose	8585	1418	11
118	GlcNAcβ1-6GalNAcα-sp3	core 6	7860	714	4
119	Manα1-2Manβ-sp4		1200	259	0
120	Manα1-3Manβ-sp4		1224	338	0
121	Manα1-4Manβ-sp4		1064	328	0
122	Manα1-6Manβ-sp4		1484	380	0
123	Manβ1-4GlcNAcβ-sp4		2103	428	2
125	6-Bn-Galβ1-4GlcNAcβ-sp2		32674	5389	19
126	6-Bn-Galα1-4(6-Bn)GlcNAcβ-sp3		15410	3185	15
127	Galβ1-4Glcβ-dipeptide		559	105	0
128	Galβ1-4Glcβ-dipeptide		1181	263	0
129	Galβ1-3(6-O-Bn)GlcNAcβ-sp3		683	184	0
130	(6-O-Bn-Galβ1)-3GlcNAcβ-sp3		378	104	0
131	(6-O-Bn-Galβ1)-3(6-O-Bn)GlcNAcβ-sp3		1908	494	2
132	Galb1-3GalNAca-sp5	TF	376	72	0
133	Galβ1-4Glcβ-dipeptide		6816	465	4
134	Galβ1-4Glcβ-dipeptide		1197	271	0
135	Galβ1-4Glcβ-dipeptide		941	330	0
136	Galβ1-4Glcβ-dipeptide		476	98	0
137	Galβ1-4Glcβ-dipeptide		589	88	0
138	Galβ1-4Glcβ-dipeptide		571	130	0
139	Galβ1-4Glcα-sp3		395	65	0
140	Galα1-3GalNAc(fur)β-sp3		608	117	0
142	GlcNAcα1-3GalNAcβ-sp3		4523	1339	4
143	Fucα1-2(3-O-Su)Galβ-sp3		468	138	0
144	Galβ1-3(6-O-Su)GlcNAcβ-sp2		297	73	0
145	Galβ1-3(6-O-Su)GlcNAcβ-sp3		388	76	0
146	Galβ1-4(6-O-Su)Glcβ-sp2		2044	469	2
149	GlcNAcβ1-4(6-O-Su)GlcNAcβ-sp2		2569	475	5
150	3-O-Su-Galβ1-3GalNAcα-sp3		37943	3232	20
151	6-O-Su-Galβ1-3GalNAcα-sp3		1840	250	2
152	3-O-Su-Galβ1-4Glcβ-sp2	SM3	2564	413	2
153	6-O-Su-Galβ1-4Glcβ-sp2		1796	504	2
154	3-O-Su-Galβ1-3GlcNAcβ-sp3		32651	3954	20
156	3-O-Su-Galβ1-4GlcNAcβ-sp2		2305	458	1
158	4-O-Su-Galβ1-4GlcNAcβ-sp2		2927	860	2

159	4-O-Su-Galβ1-4GlcNAcβ-sp3		1656	736	2
160	6-O-Su-Galβ1-3GlcNAcβ-sp2		346	142	0
161	6-O-Su-Galβ1-3GlcNAcβ-sp3		536	141	0
162	6-O-Su-Galβ1-4GlcNAcβ-sp2		5615	1540	7
164	GlcAβ1-3GlcNAcβ-sp3		979	218	1
165	GlcAβ1-3Galβ-sp3		1035	308	1
166	GlcAβ1-6Galβ-sp3		39105	2993	17
167	GlcNAcβ1-4-[HOOC(CH_3)CH]-3-O-GlcNAcβ-sp4	GlcNAc-Mur	1012	269	1
168	GlcNAcβ1-4Mur-L-Ala-D-i-Gln-Lys	GMDP-Lys	3668	152	1
169	Neu5Acα2-3Galβ-sp3	GM4	2305	596	3
170	Neu5Acα2-6Galβ-sp3		4157	633	3
171	Neu5Acα2-3GalNAcα-sp3	3-SiaT$_n$	844	168	1
172	Neu5Acα2-6GalNAcα-sp3	SiaT$_n$	3250	124	1
173	Neu5Acβ2-6GalNAcα-sp3	b-SiaTn	3221	855	5
174	Neu5Gcα2-6GalNAcα-sp3	Neu5Gc-T$_n$	1596	178	1
175	Neu5Gcβ2-6GalNAcα-sp3		1453	451	2
176	3-O-Su-Galβ1-4(6-O-Su)Glcβ-sp2		43008	9342	20
177	3-O-Su-Galβ1-4(6-O-Su)GlcNAcβ-sp3		32496	7215	20
178	6-O-Su-Galβ1-4(6-O-Su)Glcβ-sp2		3506	763	5
179	6-O-Su-Galβ1-3(6-O-Su)GlcNAcβ-sp2		1770	580	0
180	6-O-Su-Galβ1-4(6-O-Su)GlcNAcβ-sp2		2991	832	4
181	3,4-O-Su$_2$-Galβ1-4GlcNAcβ-sp3		2993	820	5
182	3,6-O-Su$_2$-Galβ1-4GlcNAcβ-sp2		487	151	0
183	4,6-O-Su$_2$-Galβ1-4GlcNAcβ-sp2		4401	944	4
184	4,6-O-Su$_2$-Galβ1-4GlcNAcβ-sp3		1945	649	2
186	Neu5Acα2-8Neu5Acα2-sp3	(Sia)$_2$	190	64	0
187	3-O-Su-Galβ1-4(6-O-Su)Glcβ-sp2		290	91	0
188	Neu5Acα2-8Neu5Acβ-sp9		1162	382	2
189	3,6-O-Su$_2$-Galβ1-4(6-O-Su)GlcNAcβ-sp2		2054	543	3
190	Galβ1-4-(6-P)GlcNAcβ-sp2		1217	347	1
191	6-P-Galβ1-4GlcNAcβ-sp2		926	194	1
192	GalNAcβ1-4(6-O-Su)GlcNAcβ-sp3		483	106	0
193	3-O-Su-GalNAcβ1-4GlcNAcβ-sp3		3103	626	4
194	6-O-Su-GalNAcβ1-4GlcNAcβ-sp3		2884	455	4
195	6-O-Su-GalNAcβ1-4-(3-O-Ac)GlcNAcβ-sp3		589	122	0
196	3-O-Su-GalNAcβ1-4(3-O-Su)-GlcNAcβ-sp3		605	123	1
197	3,6-O-Su$_2$-GalNAcβ1-4-GlcNAcβ-sp3		287	54	0

198	4,6-O-Su$_2$-GalNAcβ1-4GlcNAcβ-sp3		2251	407	1
199	4,6-O-Su$_2$-GalNAcβ1-4-(3-O-Ac)GlcNAcβ-sp3		690	178	0
200	4-O-Su-GalNAcβ1-4GlcNAcβ-sp3		466	134	0
201	3,4-O-Su$_2$-GalNAcβ1-4-GlcNAcβ-sp3		3790	859	6
202	6-O-Su-GalNAcβ1-4(6-O-Su)GlcNAcβ-sp3		4471	933	8
203	Galβ1-4(6-O-Su)GlcNAcβ-sp2		5424	1203	8
204	4-O-Su-GalNAcβ1-4GlcNAcβ-sp2		753	120	1
205	Neu5Acα2-6GalNAcβ-sp3		606	111	1
206	Neu5Gcα2-3Gal-sp3		1295	371	0
207	Neu5Acβ2-6GalNAcβ-sp3		2913	646	3
208	Galβ1-3GlcNAcβ-sp4	Le^C	658	172	0
209	Crypted	Crypted	6503	1331	9
210	Crypted	Crypted	1743	558	1
211	Crypted	Crypted	8440	1533	13
212	Crypted	Crypted	2129	530	3
213	Neu5Acβ2-6Galβ-sp3		2672	827	3
214	ΔGlcAβ1-3Galβ-sp3	deltaGlcAβ3Gal	589	161	0
215	Fucα1-2Galβ1-3GlcNAcβ-sp3	Le^d, H (type 1)	2727	444	3
216	Fucα1-2Galβ1-4GlcNAcβ-sp3	H (type 2)	10845	2895	15
217	Fucα1-2Galβ1-3GalNAcα-sp3	H (type 3)	3108	468	6
219	Fucα1-2Galβ1-4Glcβ-sp4	H (type 6)-Gly	8251	1687	10
220	Galα1-3Galβ1-4Glcβ-sp2		901	275	0
221	Galα1-3Galβ1-4Glcβ-sp4		1230	393	2
222	Galα1-3Galβ1-4GlcNAcβ-sp3	Galili (tri)	254	106	0
223	Galα1-4Galβ1-4Glcβ-sp2	P^k, Gb3, $GbOse_3$	756	247	0
225	Galα1-4Galβ1-4GlcNAcβ-sp2	P_1	573	165	0
226	Galα1-3(Fucα1-2)Galβ-sp3	B_{tri}	1227	312	1
227	Galα1-3(Fucα1-2)Galβ-sp5	B_{tri}-C8	1044	248	1
228	Galβ1-2Galα1-3GlcNAcβ-sp3		321	87	0
229	Galβ1-3Galβ1-4GlcNAcβ-sp4		323	81	0
230	Galβ1-4Galβ1-4Glcβ-sp2	Mc3	248	84	0
232	Galβ1-4GlcNAcβ1-6GalNAcα–sp3		624	81	1
233	Galβ1-3(Fucα1-4)GlcNAcβ-sp3	Le^A	210	58	0
234	Galβ1-4(Fucα1-3)GlcNAcβ-sp3	Le^X	13573	2635	16
235	GalNAcα1-3(Fucα1-2)Galβ-sp3	A_{tri}	586	306	1
236	GalNAcα1-3(Fucα1-2)Galβ-sp5	A_{tri}	498	262	1

237	GalNHα1-3<br>Galβ-$OCH_2CH_2CH_2NHAc$<br>Fucα1-2		357	135	0
239	GalNAcβ1-3(Fucα1-2)Galβ-sp3		849	654	1
240	(Glcα1-4)$_3$β-sp4	maltotriose	11233	1567	6
241	(Glcα1-6)$_3$β-sp4	isomaltotriose	520	163	0
242	GlcNAcα1-3Galβ1-4GlcNAcβ-sp2		278	90	0
243	GlcNAcα1-3Galβ1-4GlcNAcβ-sp3		215	61	0
245	GlcNAcα1-6Galβ1-4GlcNAcβ-sp2		3394	1053	3
246	GlcNAcβ1-2Galβ1-3GalNAcα-sp3		434	94	0
247	GlcNAcβ1-3Galβ1-3GalNAcα-sp3		321	63	0
248	GlcNAcβ1-3Galβ1-4Glcβ-sp2		822	179	0
249	GlcNAcβ1-3Galβ1-4GlcNAcβ-sp2		1652	322	2
250	GlcNAcβ1-3Galβ1-4GlcNAcβ-sp3		1942	488	2
251	GlcNAcβ1-4Galβ1-4GlcNAcβ-sp2		1067	216	1
252	GlcNAcβ1-4GlcNAcβ1-4GlcNAcβ-sp4	chitotriose	1111	206	2
253	GlcNAcβ1-6Galβ1-4GlcNAcβ-sp2		2877	653	3
254	GlcNAcβ1-6(Galβ1-3)GalNAcα-sp3	core 2	2499	525	4
255	GlcNAcβ1-6(GlcNAcβ1-3)GalNAcα-sp3	core 4	5853	425	4
256	GlcNAcβ1-6(GlcNAcβ1-4)GalNAcα-sp3		5020	613	8
258	Manα1-6(Manα1-3)Manβ-sp4	Man_3	743	160	0
259	Galβ1-4(Galβ1-3)GlcNAcβ-sp3		372	58	0
260	Fucβ1-4<br>GlcNAcβ-sp3<br>Galβ1-3		663	140	0
261	Fucβ1-3<br>GlcNAcβ-sp3<br>Galβ1-4		299	62	0
262	Galβ1-3GalNAcβ1-3Gal-sp4		437	120	0
263	(GalNAcβ-PEG2)3-β-DD		657	114	0
264	Galβ1-4Galβ1-4GlcNAcβ-sp3		461	87	0
266	Galα1-4Galβ1-4GlcNAcβ-sp3	P_1	1017	227	0
267	GlcNAcβ1-3Galβ1-3GlcNAcβ-sp3		718	138	0
268	GlcNAcβ1-4(Fucα1-6)GlcNAcβ-sp3		1291	311	1
269	Galβ1-3Galβ1-4Glcβ-sp4		518	137	0
270	Galβ1-4Galβ1-4Glcβ-sp4		939	202	0
271	Galβ1-6Galβ1-4Glcβ-sp4		53622	1934	20

272	Neu5Acα2-3Galβ1-4Glcβ-sp4-Cit	3'-SL -citrullin	236	60	0
273	Fucβ1-2Galβ1-4GlcNAcβ-sp3		242	60	0
274	GalNAcα1-3Galβ1-4GlcNAc-sp3		426	90	0
275	GalNAcβ1-3Galβ1-4GlcNAc-sp3		403	103	0
276	GlcNAcβ1-4Galβ1-4GlcNAcβ-sp3		1357	456	2
277	GalNGcα1-3(Fucα1-2)Galβ-sp3	N-Gc-A_{tri}	375	99	0
287	3-O-Su-Galβ1-3(Fucα1-4)GlcNAcβ-sp3	3'-OSu-Le^{A}	20063	4962	19
288	3-O-Su-Galβ1-4(Fucα1-3)GlcNAcβ-sp3	3'-OSu-Le^{X}	593	146	0
289	Neu5Acα2-6(Galβ1-3)GalNAcα-sp3		296	88	0
290	Neu5Acα2-6(Galα1-3)GalNAcα-sp3		262	68	0
291	Neu5Acβ2-6(Galβ1-3)GalNAcα-sp3		1887	727	3
292	Neu5Acα2-3Galβ1-3GalNAcα-sp3		4477	1186	4
293	Neu5Acα2-3Galβ1-4Glcβ-sp3	3'SL	1684	349	1
294	Neu5Acα2-3Galβ1-4Glcβ-sp4	3'SL	363	99	0
295	Neu5Acα2-6Galβ1-4Glcβ-sp2	6'SL	290	66	0
296	Neu5Acα2-6Galβ1-4Glcβ-sp4	6'SL	360	96	0
297	Neu5Acβ2-6Galβ1-4Glcβ-sp2		1645	409	3
298	Neu5Acα2-3Galβ1-4GlcNAcβ-sp3	3'SLN	5486	1855	3
299	Neu5Acα2-3Galβ1-3GlcNAcβ-sp3	3'SiaLe^{C}	3968	1266	6
300	Neu5Acα2-6Galβ1-4GlcNAcβ-sp3	6'SLN	936	527	1
302	Neu5Acβ2-6Galβ1-4GlcNAcβ-sp3		1100	368	2
303	Neu5Gcα2-3Galβ1-4GlcNAcβ-sp3		2147	451	1
304	Neu5Gcα2-6Galβ1-4GlcNAcβ-sp3		792	191	0
305	Neu5Gcβ2-6Galβ1-4GlcNAcβ-sp3		601	112	0
306	9-NAc-Neu5Acα2-6Galβ1-4GlcNAcβ-sp3		1431	437	1
307	KDNα2-3Galβ1-3GlcNAcβ-sp2		1441	304	2
308	KDNα2-3Galβ1-4GlcNAcβ-sp2		903	204	0
309	Neu5Acα2-6(Neu5Acα2-3)GalNAcα-sp3		487	107	1
310	3'SiaLacNAcβ-$OCH_2CH_2CH_2NH$-(3'SiaLacNAc-amide-sp3)		1932	665	1
315	Neu5Acα2-3Galβ1-4-(6-O-Su)GlcNAcβ-sp3		1930	477	3
316	Neu5Acα2-3Galβ1-3-(6-O-Su)GlcNAcβ-sp3		2752	1014	5
318	Neu5Acα2-6Galβ1-4-(6-O-Su)GlcNAcβ-sp3		1004	234	1
319	Neu5Acα2-3-(6-O-Su)Galβ1-4GlcNAcβ-sp3		7963	705	7
320	4-O-Su-Neu5Acα2-3-(6-O-Su)Galβ1-4GlcNAcβ-sp3		9621	1580	10
321	$(Neu5Ac\alpha2\text{-}8)_3$-sp3	$(Sia)_3$	226	57	0

322	(Neu5Acα2-8)3β-sp3		553	112	0
323	Neu5Acα2-6Galβ1-3GlcNAc-sp3	6'-SiaLe^{c}	317	100	0
324	Neu5Acα2-6Galβ1-3(6-O-Su)GlcNAc-sp3		361	80	0
325	Neu5Acα2-3Galβ1-4Glcβ-dipeptide		275	57	0
326	Neu5Acα2-3Galβ1-4Glcβ-dipeptide		311	65	0
327	Neu5Acα2-3Galβ1-4Glcβ-dipeptide		495	125	0
328	Neu5Acα2-3Galβ1-4Glcβ-dipeptide		298	55	0
329	Neu5Acα2-3Galβ1-4Glcβ-dipeptide		586	133	0
331	Neu5Gcα2-3Galβ1-3GlcNAcβ-sp3		2552	467	5
332	Neu5Acα2-3Galβ1-4Glcβ-dipeptide		381	91	0
333	Neu5Gcα2-3Galβ1-3-(6-O-Su)GlcNAcβ-sp3		4811	1109	5
334	Neu5Gcα2-3Galβ1-4-(6-O-Su)GlcNAcβ-sp3		1639	274	2
335	Neu5Acα2-3Galβ1-3-(6-O-Su)GlcNAcβ-sp3		6401	1102	6
336	αKdo-(2→8)-αKdo-(2→4)-αKdo-sp11		5921	1649	10
337	GalNAcα1-4Galβ1-4GlcNAcβ-sp3		4219	657	7
338	Neu5Acα2-6Galβ1-3GalNAcα-sp3		306	59	0
339	Neu5Acβ2-6Galβ1-3GalNAcα-sp3		1931	365	3
340	Galα1-3(Neu5Acβ2-6)GalNAcβ-sp3		2907	760	4
341	Neu5Acα2-3-(6-Su)Galβ1-4GlcNAcβ-sp2		5964	1255	9
359	Galα1-3(Fucα1-2)Galβ1-3GlcNAcβ-sp3	B (type 1)	400	112	0
360	Galα1-3(Fucα1-2)Galβ1-4GlcNAcβ-sp3	B (type 2)	5071	644	6
362	Galα1-3(Fucα1-2)Galβ1-3GalNAcα-sp3	B (type 3)	8283	2150	14
363	Galα1-3(Fucα1-2)Galβ1-3GalNAcβ-sp3	B (type 4)	2728	589	6
364	Galα1-3Galβ1-4(Fucα1-3)GlcNAcβ-sp3	αGalLe^{X}	541	116	0
365	Galα1-4(Fucα1-2)Galβ1-4GlcNAcβ-sp3		962	361	1
366	GalNAcα1-3(Fucα1-2)Galβ1-3GlcNAcβ-sp3	A (type 1)	351	71	0
368	GalNAcα1-3(Fucα1-2)Galβ1-4GlcNAcβ-sp3	A (type 2)	2621	625	2
369	GalNAcα1-4(Fucα1-2)Galβ1-4GlcNAcβ-sp3		3246	374	3
370	Crypted	Crypted	2629	835	3
371	Fucα1-2Galβ1-3(Fucα1-4)GlcNAcβ-sp3	Le^{B}	307	107	0
372	Fucα1-2Galβ1-4(Fucα1-3)GlcNAcβ-sp3	Le^{Y}	3936	1040	8
373	Galα1-3Galβ1-4GlcNAcβ1-3Galβ-sp3	Galili (tetra)	777	128	0
374	Galα1-3(Galα1-4)Galβ1-4GlcNAcβ-sp3	Galα2-3',4'LN	280	72	0
375	Galα1-4GlcNAcβ1-3Galβ1-4GlcNAcβ-sp3		1032	190	1
377	Galβ1-3GlcNAcβ1-3Galβ1-3GlcNAcβ-sp2		827	166	1
378	Galβ1-3GlcNAcα1-3Galβ1-4GlcNAcβ-sp3		10106	2009	9
379	Galβ1-3GlcNAcβ1-3Galβ1-4GlcNAcβ-sp3		1304	387	1

380	Galβ1-3GlcNAca1-6Galb1-4GlcNAcb-sp2		2306	344	1
381	Galβ1-3GlcNAcβ1-6Galβ1-4GlcNAcβ-sp2		2581	827	3
382	Galβ1-3GalNAcβ1-4Galβ1-4Glcβ-sp3	GA1, asialo-GM1	642	125	0
383	Galβ1-4GlcNAcβ1-3Galβ1-4Glcβ-sp4	LNnT	280	74	0
384	Galβ1-4GlcNAcβ1-3Galβ1-4GlcNAcβ-sp2	i	290	76	0
386	Galβ1-4GlcNAcα1-6Galβ1-4GlcNAcβ-sp2		3822	638	2
387	Galβ1-4GlcNAcβ1-6Galβ1-4GlcNAcβ-sp2		2719	521	2
388	Galβ1-4GlcNAcβ1-6(Galβ1-3)GalNAcα-sp3		229	77	0
389	GalNAcβ1-3Galα1-4Galβ1-4Glcβ-sp3	Gb4, P	399	90	0
390	(Glcα1-4)$_4$β-sp4	maltotetraose	13504	2947	10
394	GlcNAcβ1-4				
	Galβ1-4GlcNAcβ-sp2		2286	487	3
	GlcNAcβ1-3				
395	GlcNAcβ1-6(GlcNAcβ1-3)Galβ1-4GlcNAcβ-sp2	T_k	1249	316	0
396	(GlcNAcβ1)3-3,4,6-GalNAcα-sp3		4966	727	4
397	(GlcNAcβ1)$_3$-3,4,6-GalNAcα-sp3		7793	1480	9
398	Galβ1-3GlcN(Fm)β1-3Galβ1-4GlcNAcβ-sp3		557	106	0
399	Galβ1-3GlcNAcα1-3Galβ1-3GlcNAcβ-sp2		6788	1178	10
401	Galβ1-3GlcNAcβ1-3Galβ1-3GlcNAcβ-sp3		717	181	0
402	GalNAcα1-3				
	Galα1-4GlcNAcβ-sp3		175	45	0
	Fucα1-2				
403	Galβ1-3GlcNAcβ1-3Galβ1-4GlcNAcβ-sp2		2047	376	4
404	GalNAcα1-3Galβ1-4(Fucα1-3)GlcNAcβ-sp3	αGalNAcLe^X	3611	1529	5
405	Galα1-3(Fucα1-2)Galα1-4GlcNAcβ-sp3		2929	227	1
406	GalNAcα1-3(Fucα1-2)Galα1-3GalNAcβ-sp3		304	60	0
419	3-O-SuGalβ1-4GlcNAcβ1-3Galβ1-4GlcNAcβ-sp3		2425	448	3
420	4-O-SuGalβ1-4GlcNAcβ1-3Galβ1-4GlcNAcβ-sp3		894	183	1
423	Neu5Acα2-3Galβ1-4(Fucα1-3)GlcNAcβ-sp3	SiaLe^X	1673	483	1
425	Neu5Acα2-3Galβ1-4				
	GlcNAcβ-sp3		2164	673	2
	Fucβ1-3				
426	Neu5Acα2-3Galβ1-3(Fucα1-4)GlcNAcβ-sp3	SiaLe^A	216	60	0
428	Neu5Acα2-3Galβ1-4(Fucα1-3)(6-O-Su-)GlcNAcβ-sp3	6-OSu-SLe^x	429	149	0

429	Neu5Acα2-3(6-O-Su)Galβ1-4(Fucα1-3)GlcNAcβ-sp3	6’-OSu-SiaLe^{x}	762	222	2
431	Neu5Acα2-3Galβ1-4(2-O-Su-Fucα1-3)GlcNAcβ-sp3		918	242	2
432	Neu5Acα2-3Galβ1-4<br>GlcNAcβ-sp3<br>3-O-Su-Fucα1-3		546	207	0
433	Neu5Acα2-6(Neu5Acα2-3Galβ1-3)GalNAcα-sp3		203	66	0
434	Neu5Acα2-8Neu5Acα2-3Galβl-4Glcβ-sp4	GD3	287	84	0
435	Neu5Acα2-3Galβ1-4(2-O-Su-Fucα1-3)(6-O-Su-)GlcNAcβ-sp3		425	133	0
436	4-O-Su-Neu5Acα2-3Galβ1-4(Fucα1-3)(6-O-Su-)GlcNAcβ-sp3		963	415	2
437	GalNAcα1-3(Fucα1-2)Galβ1-3GalNAcβ-sp3	A(type 4)	33886	3193	18
438	Fucβ1-2Galβ1-4(Fucα1-3)GlcNAcβ-sp3		460	131	0
439	αKdo-(2→4)-αKdo-(2→4)-αKdo-(2→6)-βGlcNAc-sp11		5179	1212	9
440	Neu5Acβ2-6(Fucα1-2)Galβ1-4GlcNAcβ-sp3		2150	416	3
441	Neu5Acα2-6(Fucα1-2)Galβ1-4GlcNAcβ-sp3		719	160	1
442	Neu5Acα2-3 (GalNAcβ1-4)Galβ1-4Glcβ-sp4	GM2	499	111	0
479	Fucα1-2Galβ1-3GlcNAcβ1-3Galβ1-4Glcβ-sp4	LNFP I	512	286	0
480	Fucα1-2Galβ1-3GlcNAcβ1-3Galβ1-4GlcNAcβ-sp2	H(type 1) penta	3826	911	8
481	Galα1-3Galβ1-4GlcNAcβ1-3Galβ1-4Glcβ-sp4	Galili (penta)	241	60	0
482	Galα1-3(Fucα1-2)Galβ1-3(Fucα1-4)GlcNAcβ-sp3	BLe^{B}	1726	229	3
483	Galα1-3(Fucα1-2)Galβ1-4(Fucα1-3)GlcNAcβ-sp3	BLe^{Y}	2689	293	3
484	GalNAcα1-3(Fucα1-2)Galβ1-3(Fucα1-4)GlcNAcβ-sp3	ALe^{B}	175	49	0
485	Galβ1-4GalNAcα1-3(Fucα1-2)Galβ1-4GlcNAcβ-sp3		2643	642	4
488	Galβ1-4GlcNAcβ1-6(Galβ1-4GlcNAcβ1-3)GalNAcα-sp3		284	69	0
489	Galβ1-4GlcNAcβ1-3(GlcNAcβ1-6)Galβ1-4GlcNAcβ-sp2		541	172	0
490	Galβ1-4GlcNAcβ1-6(GlcNAcβ1-3)Galβ1-4GlcNAcβ-sp2		527	119	0
491	GalNAcα1-3(Fucα1-2)Galβ1-4(Fucα1-3)GlcNAcβ-sp3	ALe^{Y}	213	65	0
492	(Glcα1-6)$_5$β-sp4	isomaltopentaose	1151	263	2
493	(GlcNAcβ1-4)$_5$β-sp4	chitopentaose	1798	509	1
495	Manα1-6(Manα1-3)Manα1-6(Manα1-3)Manβ-sp4	Man$_5$	892	319	1

496	Fucα1-2Galβ1-3(Fucα1-4)GlcNAcβ1-3Galβ1-4Glcβ-sp4	Le^b(hexa)	223	72	0
497	Fucα1-2Galβ1-4(Fucα1-3)GlcNAcβ1-3Galβ1-4Glcβ-sp4	Le^Y(hexa)	2057	212	4
498	Galβ1-4GlcNAcβ1-3Galβ1-4GlcNAcβ1-3Galβ1-4GlcNAcβ-sp3	$(LN)_3$	201	62	0
499	Galβ1-4GlcNAcβ1-6(Galβ1-4GlcNAcβ1-3)Galβ1-4GlcNAcβ-sp2	I	200	45	0
501	Galβ1-3GalNAcβ1-3Galα1-4Galβ1-4Glcβ-sp4	Gb5	1360	254	1
502	$(Glcα1\text{-}6)_6$β-sp4	maltohexaose	3033	653	5
503	(GlcNAcβ1-4)6-sp4	chitohexaose	2396	314	1
504	$(A\text{-}GN\text{-}M)_2$-3,6-M-GN-GNβ-sp4	9-OS	200	57	0
505	$(GN\text{-}M)_2$-3,6-M-GN-GNβ-sp4	7-OS	728	195	0
506	Araf6	Ara6	158	37	0
507	GalNAcα1-3GalNAcβ1-3Galα1-4Galβ1-4Glcβ-sp3	Fs-5	2021	988	3
508	GalNAcβ1-3(Fucα1-2)Galβ1-4(Fucα1-3)GlcNAcβ-sp3		402	93	0
509	Galβ1-3GalNAcβ1-4(Neu5Acα2-3)Galβ1-4Glcβ-sp4	GM1	1553	448	3
527	Neu5Acα2-3Galβ1-4GlcNAcβ1-3Galβ1-4GlcNAcβ-sp2		179	48	0
528	Neu5Acα2-3Galβ1-4(Fucα1-3)GlcNAcβ1-3Galβ-sp3	$SiaLe^x$-3Gal	203	49	0
529	Neu5Acα2-6(Galβ1-3)GlcNAcβ1-3Galβ1-4Glcβ-sp4	LSTb	297	70	0
530	(Neu5Acα2-3Galβ1)2-3,4-GlcNAcβ-sp3		1529	352	1
531	Neu5Acα2-8Neu5Acα2-3(GalNAcβ1-4)Galβ1-4Glcβ-sp2	GD2	434	116	0
534	Neu5Acα2-3Galβ1-4GlcNAcβ1-3Galβ1-4GlcNAcβ-sp3		193	58	0
535	Neu5Acα2-8Neu5Acα2-3(GalNAcβ1-4)Galβ1-4Glc-sp4	GD2	234	61	0
536	Neu5Aα2-3Galβ1-3GlcNAcβ1-3Galβ1-4Glcβ-sp4	LSTa	342	92	0
537	Neu5Aα2-3Galβ1-4GlcNAcβ1-3Galβ1-4Glcβ-sp4	LSTd	202	61	0
538	Galβ1-4(Fucα1-3)GlcNAcβ1-6(Galβ1-3GlcNAcβ1-3)Galβ1-4Glcβ-sp4	MFLNH III	2473	712	3
539	Galβ1-4GlcNAcβ1-6(Fucα1-2Galβ1-3GlcNAcβ1-3)Galβ1-4Glcβ-sp4	MFLNH I	780	239	0
540	Galβ1-4(Fucα1-3)GlcNAcβ1-6(Neu5Ac2-6Galβ1-4GlcNAcβ1-3)Galβ1-4Glcβ-sp4	MSMFLNnH	2262	337	3

541	Galβ1-4(Fucα1-3)GlcNAcβ1-6(Fucα1-2Galβ1-3GlcNAcβ1-3)Galβ1-4Glcβ-sp4	DFLNH (a)	6347	878	7
542	Galβ1-3GlcNAcβ1-3Galβ1-4(Fucα1-3)GlcNAcβ1-6(Galβ1-3GlcNAcβ1-3)Galβ1-4Glcβ-sp4	MF(1-3)iLNO	2867	344	6
543	Fucα1-2Galβ1-3(Fucα1-4)GlcNAcβ1-3[Galβ1-4(Fucα1-3)GlcNAcβ1-6]Galβ1-4Glcβ-sp4	TFLNH	3573	657	6
545	αKdo-(2→8)-αKdo-(2→4)-αKdo-(2→6)-βGlcNAc-(1→6)αGlcNAc-sp11		3329	757	6
625	(GlcAβ1-4GlcNAcβ1-3)$_{11\text{-}12}$-NH_2-ol	hyaluronic acid	776	257	1
627	(Sia2-6A-GN-M)$_2$-3,6-M-GN-GNβ-sp4	11-OS, YDS	184	54	0
629	Trehalose-ethanolamine	**negative control**	323	71	0
630	(GlcAβ1-3GlcNAcβ1-4)$_{20}$-NH(*p*-C_6H_4)$CH_2CH_2NH_2$	hyaluronic acid , 8kDa	921	236	1
631	(GlcAβ1-3GlcNAcβ1-4)$_{38}$-NH(*p*-C_6H_4)$CH_2CH_2NH_2$	hyaluronic acid, 15-30kDa	456	174	1
632	(GlcAβ1-3GlcNAcβ1-4)$_{13}$-NH(*p*-C_6H_4)$CH_2CH_2NH_2$	hyaluronic acid, 4-8kDa	481	109	0
800	GlcNAcα1-4GlcNAcβ-sp3		1697	257	1
801	GalNAcα1-3GalNAc(fur)β-sp3		3988	289	2
802	Galβ1-3GalNAc(fur)β-sp3		51348	2324	20
804	[Galβ1-4GlcNAcβ-OCH_2CH_2]$_2$NH	LN dimer	908	252	1
805	GalNAcβ1-4(6-O-Bn)GlcNAcβ-sp3		6754	1034	11
806	Galα1-6Glcα-sp3	α-melibiose	5469	949	4
807	GlcNAcβ1-4GlcNAcα-sp4		10158	984	8
808	Galα1-6Glcβ-sp3	melibiose	9724	1131	7
809	GalNAcβ1-3GalNAcα-sp3		8521	1509	13
810	GalNGcα1-3GalNAcα-sp3	core 5(Gc)	328	76	0
850	Galβ1-3(6-O-Su)GalNAcα-sp3	6-SuTF	341	102	0
851	Galα1-3(6-O-Su)GalNAcα-sp3	6-Su$T_{\alpha\alpha}$	267	86	0
852	GlcNAcβ1-4-[HOOC(CH3)CH]-3-O-GlcNAcα-sp4	GlcNAc-Murα	1056	322	1
900	H-(Gly)$_6$-NH_2 Gly6-amide, linear	Gly$_6$-amide, linear	25036	2262	16
901	biot-CMG$_2$-NH2	**biot-CMG$_2$**	52213	3728	20
902	Peptide, crypted		5630	1228	6
903	Peptide, crypted		2617	897	4
904	Peptide, crypted		6111	1154	12
905	Suc-LPWYRAPK-NH2	RhD	16175	3630	20
906	Peptide, crypted		475	163	0
907	Peptide, crypted		457	93	0
908	Peptide, crypted		6687	2162	13
909	Peptide, crypted		812	166	1

910	Npentyl-Indol-linked-3-NH2 (AcOH salt)		686	168	0
911	N-C5-indazole-3-CONH-PEG-NH2 (AcOH salt)		6037	1212	11

sp2 = $-O(CH_2)_2NH_2$

sp3 = $-O(CH_2)_3NH_2$

sp4 = $-NHCOCH_2NH_2$

sp5 = $-O(CH_2)_3NH-CO(CH_2)_5NH_2$

sp8 = $-(OCH_2CH_2)_6NH_2$

Table S2. List of glycans, their binding to human circulating IgM and IgG antibodies (n=11), expressed in relative fluorescence units (RFU) as median, and the number of human donors exceeding *cut off* (≥ 4000 RFU).

Glycan ID (#)	Structure	Common Name	IgG		IgM	
			Median	≥4000RFU	Median	≥4000RFU
001	Fucα-sp3		677	0	1296	0
002	Galα-sp3		1965	1	2752	3
003	Galβ-sp3		1466	0	7190	5
004	GalNAcα1-OSer	T_n-Ser	1185	0	2657	2
005	GalNAcα-sp3	T_n	1332	0	6289	5
006	GalNAcβ-sp3		4194	2	3882	4
007	Glcα-sp3		1168	0	1139	1
009	Glcβ-sp3		1062	0	2063	1
010	GlcNAcβ-sp3		1966	1	8507	6
011	GlcNAcβ-sp2		1608	1	6700	6
012	GlcNAcβ-sp7		1340	0	1928	1
013	GlcNAcβ-sp8		2628	1	6422	7
014	GlcN(Gc)β-sp4		904	0	1840	1
015	$HOCH_2(HOCH)_4CH_2NH_2$	aminoglucitol	265	0	484	0
016	Manα-sp3		306	0	664	1
017	Manα-sp4		1341	1	1741	1
018	Manβ-sp4		761	0	1526	1
019	ManNAcβ-sp4		9491	10	15659	10
020	Rhaα-sp3		3789	4	24247	9
021	Galβ-sp4		1937	2	1794	1
022	GlcNAcβ-sp4		845	0	708	0
023	GalNAcβ-sp4		931	0	1968	1
024	GlcNAcα-sp3		6362	11	7471	7
025	GalNAcβ-sp10		6646	3	4555	4
026	Rhaβ-sp4		7144	3	9334	5
027	3,6-O-Me_2-Glcβ-sp3	DMG	2903	2	5166	4
028	Xylβ-sp4		846	0	1315	1
029	Fucβ-sp4		965	0	2059	2
030	Glcβ-sp4		1104	0	1900	1
031	L-Araα-sp4		1167	0	1622	1
032	GalN(Gc)β-sp3		840	0	2565	3
037	3-O-Su-Galβ-sp3		845	0	805	0

038	3-O-Su-GalNAcβ-sp3		2656	3	2252	2
041	6-O-Su-GalNAcα-sp3		2630	1	7916	6
043	6-O-Su-GlcNAcβ-sp3		4577	1	5463	5
044	GlcAα-sp3	α-glucuronic acid	1237	0	2251	2
045	GlcAβ-sp3	β-glucuronic acid, β-glucuronate	701	0	2263	1
046	6-H_2PO_3Glcβ-sp4	Glc6P	1058	0	2567	2
047	6-H_2PO_3Manα-sp3	Man6P	838	0	1269	1
048	Neu5Acα-sp3		549	0	693	0
049	Neu5Acα-sp9		744	0	1206	1
050	Neu5Acβ-sp3		913	0	3326	3
051	Neu5Acβ-sp9		1209	0	4618	4
052	Neu5Gcα-sp3		999	0	1080	1
053	Neu5Gcβ-sp3		995	0	1965	1
054	9-NAc-Neu5Acα-sp3		547	0	2680	2
055	3-O-Su-GlcNAcβ-sp3		5104	4	10286	8
056	Galα-sp7		1609	1	3453	3
057	D-Ribβ -sp4		953	0	1947	1
058	Fucβ-sp3		1346	0	3018	1
059	αKdo-5-phosphate-sp11		2150	2	2808	3
060	6-O-Su-Galβ-sp3		2049	0	10416	8
061	3-O-Su-GalNAcα-sp3		1835	2	3892	5
071	Fucα1-2Galβ-sp3	H_{di}	1313	0	5610	5
072	Fucα1-3GlcNAcβ-sp3		1972	0	7528	7
073	Fucα1-4GlcNAcβ-sp3	Le	4179	5	10882	6
074	Fucβ1-3GlcNAcβ-sp3		2502	1	11474	7
075	Galα1-2Galβ-sp3		7844	7	6652	7
076	Galα1-3Galβ-sp3	B_{di}	2044	1	2442	2
077	Galα1-3GalNAcβ-sp3		3624	2	7263	6
078	Galα1-3GalNAcα-sp3	$T_{\alpha\alpha\text{, core 8}}$	1168	0	5643	4
080	Galα1-3GlcNAcβ-sp3		17986	9	14484	9
081	Galα1-4GlcNAcβ-sp3	aLN	12835	8	9160	7
082	Galα1-4GlcNAcβ-sp8	αLN	15126	8	9476	8
083	Galα1-6Glcβ-sp4	melibiose	5942	7	4403	3

084	Galβ1-2Galβ-sp3		4605	4	5243	4
085	Galβ1-3GlcNAcβ-sp3	Le^{c}	2860	2	9867	7
086	Galβ1-3GlcNAcβ-sp2	Le^{c}	3009	2	9213	6
087	Galβ1-3Galβ-sp3		1625	1	4885	4
088	Galβ1-3GalNAcβ-sp3	$T_{\beta\beta}$	4769	5	5868	5
089	Galβ1-3GalNAcα-sp3	TF, core 1	1259	1	2292	4
092	Galβ1-4Glcβ-sp2	Lactose, Lac	1488	0	1608	1
093	Galβ1-4Glcβ-sp4	Lactose, Lac	14076	9	4614	5
094	Galβ1-4Galβ-sp4		1343	1	3186	2
096	Galβ1-4GlcNAcβ-sp2	N-acetyllactosamine, LN	687	0	546	0
097	Galβ1-4GlcNAcβ-sp3	N-acetyllactosamine, LN	757	0	601	0
098	Galβ1-4GlcNAcβ–sp5	N-acetyllactosamine, LN	975	0	747	0
099	Galβ1-4GlcNAcβ-sp8	N-acetyllactosamine, LN	696	0	757	0
100	Galβ1-6Galβ-sp4		4140	3	3146	4
101	GalNAcα1-3GalNAcβ-sp3	Fs-2	8924	9	15067	10
102	GalNAcα1-3Galβ-sp3	A_{di}	4766	3	13124	8
103	GalNAcα1-3GalNAcα-sp3	core 5	2423	1	17484	9
104	GalNAcβ1-3Galβ-sp3		1809	1	3131	4
105	GalNAcβ1-3GalNAcβ-sp3	para-Fs	3084	1	6100	6
106	GalNAcβ1-4GlcNAcβ-sp3	LacdiNAc	3659	1	1789	1
107	GalNAcβ1-4GlcNAcβ-sp2	LacdiNAc	1501	1	1881	2
110	Glcα1-4Glcβ-sp3	maltose	2034	1	2267	1
111	Glcβ1-4Glcβ-sp4	cellobiose	1429	1	2171	2
112	Glcβ1-6Glcβ-sp4	gentiobiose	4221	3	4103	4
113	GlcNAcβ1-3GalNAcα-sp3	core 3	2511	1	9227	8
114	GlcNAcβ1-3Manβ-sp4		2895	2	2052	1
115	GlcNAcβ1-4GlcNAcβ-Asn	chitobiose-Asn	2399	1	5255	5
116	GlcNAcβ1-4GlcNAcβ-sp3	chitobiose	6098	4	8662	7
117	GlcNAcβ1-4GlcNAcβ-sp4	chitobiose	3400	3	6934	5
118	GlcNAcβ1-6GalNAcα-sp3	core 6	1989	0	11169	7
119	Manα1-2Manβ-sp4		1260	0	1515	1
120	Manα1-3Manβ-sp4		1318	1	2124	1

121	Manα1-4Manβ-sp4		1115	0	1508	1
122	Manα1-6Manβ-sp4		1190	0	1103	1
123	Manβ1-4GlcNAcβ-sp4		1619	0	5040	6
125	6-Bn-Galβ1-4GlcNAcβ-sp2		735	0	867	0
126	6-Bn-Galα1-4(6-Bn)GlcNAcβ-sp3		11774	8	7860	7
127	Galβ1-4Glcβ-sp4-Phe		24027	10	5351	5
128	Galβ1-4Glcβ-sp4-Trp		22860	10	4697	4
129	Galβ1-3(6-O-Bn)GlcNAcβ-sp3		909	0	1027	0
130	(6-O-Bn-Galβ1)-3GlcNAcβ-sp3		4975	3	9337	8
131	(6-O-Bn-Galβ1)-3(6-O-Bn)GlcNAcβ-sp3		2880	3	2477	2
132	Galβ1-3GalNAcα-sp5	TF	1750	2	3748	4
133	Galβ1-4Glcβ-sp4-Ala		13553	9	3557	2
134	Galβ1-4Glcβ-sp4-Arg		17986	10	3894	3
135	Galβ1-4Glcβ-sp4-Asn		7428	8	2001	1
136	Galβ1-4Glcβ-sp4-Ile		24449	10	5144	4
137	Galβ1-4Glcβ-sp4-Nle		23698	11	5828	6
138	Galβ1-4Glcβ-sp4-Val		24229	11	4862	4
139	Galβ1-4GlcNAcα-sp3		7107	4	7404	6
140	Galα1-3GalNAc(fur)β-sp3		6013	5	11683	8
142	GlcNAcα1-3GalNAcβ-sp3		10447	6	15367	8
143	Fucα1-2(3-O-Su)Galβ-sp3		1360	1	3954	2
144	Galβ1-3(6-O-Su)GlcNAcβ-sp2		1397	1	2172	3
145	Galβ1-3(6-O-Su)GlcNAcβ-sp3		1141	0	3235	4
146	Galβ1-4(6-O-Su)Glcβ-sp2		1278	0	3980	2
149	GlcNAcβ1-4(6-O-Su)GlcNAcβ-sp2		13313	9	9010	9
150	3-O-Su-Galβ1-3GalNAcα-sp3		882	0	2616	2
151	6-O-Su-Galβ1-3GalNAcα-sp3		2286	2	5420	5
152	3-O-Su-Galβ1-4Glcβ-sp2	SM3	797	0	1850	2
153	6-O-Su-Galβ1-4Glcβ-sp2		3351	1	3015	3
154	3-O-Su-Galβ1-3GlcNAcβ-sp3		3261	4	9337	7
156	3-O-Su-Galβ1-4GlcNAcβ-sp2		496	0	1225	1
158	4-O-Su-Galβ1-4GlcNAcβ-sp2		1368	0	7153	4
159	4-O-Su-Galβ1-4GlcNAcβ-sp3		1266	0	6612	4
160	6-O-Su-Galβ1-3GlcNAcβ-sp2		1895	1	6993	7
161	6-O-Su-Galβ1-3GlcNAcβ-sp3		1992	1	8381	7
162	6-O-Su-Galβ1-4GlcNAcβ-sp2		618	0	1174	0

164	GlcAβ1-3GlcNAcβ-sp3		2720	1	8782	7
165	GlcAβ1-3Galβ-sp3		866	0	1408	1
166	GlcAβ1-6Galβ-sp3		1783	0	3100	2
167	GlcNAcβ1-4-[HOOC(CH_3)CH]-3-O-GlcNAcβ-sp4	GlcNAc-Mur	12596	8	6005	6
168	GlcNAcβ1-4Mur-L-Ala-D-i-Gln-Lys	GMDP-Lys	24991	8	8621	7
169	Neu5Acα2-3Galβ-sp3	GM4	738	0	1160	1
170	Neu5Acα2-6Galβ-sp3		894	0	5296	4
171	Neu5Acα2-3GalNAcα-sp3		1245	1	2614	1
172	Neu5Acα2-6GalNAcα-sp3	$SiaT_n$	6510	2	4855	4
173	Neu5Acβ2-6GalNAcα-sp3		1035	0	5472	4
174	Neu5Gcα2-6GalNAcα-sp3	Neu5GcTn	1244	0	5605	5
175	Neu5Gcβ2-6GalNAcα-sp3		980	0	3826	2
176	3-O-Su-Galβ1-4(6-O-Su)Glcβ-sp2		937	0	2054	1
177	3-O-Su-Galβ1-4(6-O-Su)GlcNAcβ-sp3		1093	1	1083	1
178	6-O-Su-Galβ1-4(6-O-Su)Glcβ-sp2		765	0	2677	2
179	6-O-Su-Galβ1-3(6-O-Su)GlcNAcβ-sp2		850	0	2060	2
180	6-O-Su-Galβ1-4(6-O-Su)GlcNAcβ-sp2		655	0	1268	0
181	3,4-O-Su_2-Galβ1-4GlcNAcβ-sp3		612	0	2829	3
182	3,6-O-Su_2-Galβ1-4GlcNAcβ-sp2		585	0	507	0
183	4,6-O-Su_2-Galβ1-4GlcNAcβ-sp2		1557	0	8861	8
184	4,6-O-Su_2-Galβ1-4GlcNAcβ-sp3		1386	0	7879	6
186	Neu5Acα2-8Neu5Acα2-sp3	$(Neu5Ac)_2$, $(Sia)_2$	315	0	285	0
187	Neu5Acα2-8Neu5Acα2-sp9	$(Sia)_2$	370	0	449	0
188	Neu5Acα2-8Neu5Acβ-sp9		649	0	5047	4
189	3,6-O-Su_2-Galβ1-4(6-O-Su)GlcNAcβ-sp2		1049	0	1989	1
190	Galβ1-4-(6-P)GlcNAcβ-sp2		831	0	1237	0
191	6-P-Galβ1-4GlcNAcβ-sp2		1814	2	4644	3
192	GalNAcβ1-4(6-O-Su)GlcNAcβ-sp3		2549	2	2059	1
193	3-O-Su-GalNAcβ1-4GlcNAcβ-sp3		5239	3	5131	5
194	6-O-Su-GalNAcβ1-4GlcNAcβ-sp3		769	0	2238	2
195	6-O-Su-GalNAcβ1-4(3-O-Ac)GlcNAcβ-sp3		820	0	1976	1
196	3-O-Su-GalNAcβ1-4(3-O-Su)GlcNAcβ-sp3		3124	3	4423	6
197	3,6-O-Su_2-GalNAcβ1-4GlcNAcβ-sp3		6226	4	4697	5
198	4,6-O-Su_2-GalNAcβ1-4GlcNAcβ-sp3		522	0	811	0
199	4,6-O-Su_2-GalNAcβ1-4-(3-O-Ac)GlcNAcβ-sp3		787	0	2264	1

200	4-O-Su-GalNAcβ1-4GlcNAcβ-sp3		457	0	633	0
201	3,4-O-Su_2-GalNAcβ1-4GlcNAcβ-sp3		2862	1	6143	7
202	6-O-Su-GalNAcβ1-4(6-O-Su)GlcNAcβ-sp3		5303	3	3194	2
203	Galβ1-4(6-O-Su)GlcNAcβ-sp2		554	0	624	0
204	4-O-Su-GalNAcβ1-4GlcNAcβ-sp2		2579	1	699	0
205	Neu5Acα2-6GalNAcβ-sp3		4506	1	3927	3
206	Neu5Gcα2-3Galβ-sp3		662	0	640	0
207	Neu5Acβ2-6GalNAcβ-sp3		904	0	4445	4
208	Galβ1-3GlcNAcβ-sp4	Le^c	1436	1	910	1
209	αKdo-(2→4)-αKdo-sp11		3676	4	7193	5
210	L,D-Hep-(1→5)-αKdo-4-phosphate-sp11		929	0	3597	3
211	αKdo-(2→8)-αKdo-sp11		5903	6	7650	6
212	D-glycero-α-D-talo-octulosonic acid (Ko)-(2→4)-αKdo-sp11		1343	1	2250	1
213	Neu5Acβ2-6Galβ-sp3		1336	0	4532	4
214	ΔGlcAβ1-3Galβ-sp3	ΔGlcAβ3Gal	739	0	1900	1
215	Fucα1-2Galβ1-3GlcNAcβ-sp3	Le^d, H (type 1)	801	0	4142	3
216	Fucα1-2Galβ1-4GlcNAcβ-sp3	H (type 2)	348	0	548	0
217	Fucα1-2Galβ1-3GalNAcα-sp3	H (type 3)	894	0	2751	2
219	Fucα1-2Galβ1-4Glcβ-sp4	H (type 6)	7996	6	2440	3
220	Galα1-3Galβ1-4Glcβ-sp2		1372	0	3201	4
221	Galα1-3Galβ1-4Glcβ-sp4		6739	7	3490	3
222	Galα1-3Galβ1-4GlcNAcβ-sp3	Galili (tri)	1875	2	3719	3
223	Galα1-4Galβ1-4Glcβ-sp2	P^k, Gb3, $GbOse_3$	2134	1	5424	6
225	Galα1-4Galβ1-4GlcNAcβ-sp2	P_1	1812	1	6721	5
226	Galα1-3(Fucα1-2)Galβ-sp3	B_{tri}	1107	0	2283	2
227	Galα1-3(Fucα1-2)Galβ-sp5	B_{tri}	1044	0	2078	2
228	Galβ1-2Galα1-3GlcNAcβ-sp3		4034	4	4644	5
229	Galβ1-3Galβ1-4GlcNAcβ-sp4		1178	0	5158	5
231	Galβ1-4GlcNAcβ1-3GalNAcα–sp3		755	0	2856	2
232	Galβ1-4GlcNAcβ1-6GalNAcα–sp3		2262	2	7259	7
233	Galβ1-3(Fucα1-4)GlcNAcβ-sp3	Le^a	558	0	788	1
234	Galβ1-4(Fucα1-3)GlcNAcβ-sp3	Le^x	424	0	345	0
235	GalNAcα1-3(Fucα1-2)Galβ-sp3	A_{tri}	3866	3	3878	2
236	GalNAcα1-3(Fucα1-2)Galβ-sp5	A_{tri}	3469	3	2384	3

237	GalNHα1-3(Fucα1-2)Galβ-OCH$_2$CH$_2$CH$_2$NHAc	AB$_{tri}$	498	0	453	0
239	GalNAcβ1-3(Fucα1-2)Galβ-sp3		819	0	3372	4
240	(Glcα1-4)$_3$β-sp4	maltotriose	1176	1	1655	2
241	(Glcα1-6)$_3$β-sp4	isomaltotriose	1125	0	1146	0
242	GlcNAcα1-3Galβ1-4GlcNAcβ-sp2		2786	3	14904	9
243	GlcNAcα1-3Galβ1-4GlcNAcβ-sp3		3035	2	16162	9
245	GlcNAcα1-6Galβ1-4GlcNAcβ-sp2		3974	3	4436	5
246	GlcNAcβ1-2Galβ1-3GalNAcα-sp3		14644	10	11352	9
247	GlcNAcβ1-3Galβ1-3GalNAcα-sp3		988	0	3707	2
248	GlcNAcβ1-3Galβ1-4Glcβ-sp2		1050	0	1930	1
249	GlcNAcβ1-3Galβ1-4GlcNAcβ-sp2		565	0	820	0
250	GlcNAcβ1-3Galβ1-4GlcNAcβ-sp3		1365	1	685	0
251	GlcNAcβ1-4Galβ1-4GlcNAcβ-sp2		7196	5	12322	7
252	GlcNAcβ1-4GlcNAcβ1-4GlcNAcβ-sp4	chitotriose	3352	2	6177	6
253	GlcNAcβ1-6Galβ1-4GlcNAcβ-sp2		1161	1	2113	1
254	GlcNAcβ1-6(Galβ1-3)GalNAcα-sp3	core 2	1163	1	2812	4
255	GlcNAcβ1-6(GlcNAcβ1-3)GalNAcα-sp3	core 4	4251	2	8235	8
256	GlcNAcβ1-6(GlcNAcβ1-4)GalNAcα-sp3		12212	10	16124	10
258	Manα1-6(Manα1-3)Manβ-sp4	Man$_3$	916	0	1610	1
259	Galβ1-4(Galβ1-3)GlcNAcβ-sp3		589	0	741	1
260	Galβ1-3(Fucβ1-4)GlcNAcβ-sp3		544	0	472	0
261	Galβ1-4(Fucβ1-3)GlcNAcβ-sp3		1066	1	611	0
262	Galβ1-3GalNAcβ1-3Galβ-sp4		3215	3	5652	4
263	(GalNAcβ-PEG$_2$)$_3$-β-DD		7288	2	7754	7
264	Galβ1-4Galβ1-4GlcNAcβ-sp3		5559	5	11786	8
266	Galα1-4Galβ1-4GlcNAcβ-sp3	P$_1$	1416	0	5773	5
267	GlcNAcβ1-3Galβ1-3GlcNAcβ-sp3		2536	3	11891	7
268	GlcNAcβ1-4(Fucα1-6)GlcNAcβ-sp3		2776	1	5150	6
269	Galβ1-3Galβ1-4Glcβ-sp4		5049	5	5410	5
270	Galβ1-4Galβ1-4Glcβ-sp4		1923	1	5551	4
271	Galβ1-6Galβ1-4Glcβ-sp4		2703	3	6145	6
272	Neu5Acα2-3Galβ1-4Glcβ-sp4-Cit		10986	6	1191	1
273	Fucβ1-2Galβ1-4GlcNAcβ-sp3		795	0	3725	2
274	GalNAcα1-3Galβ1-4GlcNAcβ-sp3		4604	2	13900	8
275	GalNAcβ1-3Galβ1-4GlcNAcβ-sp3		1060	0	2174	2
276	GlcNAcβ1-4Galβ1-4GlcNAcβ-sp3		8309	6	10740	7

277	GalN(Gc)α1-3(Fucα1-2)Galβ-sp3	A_{tri}(Gc)	1220	2	762	0
278	GalNAcα1-3GalNAcβ1-3Galβ-sp3	Fs-3	8792	8	19525	9
287	3-O-Su-Galβ1-3(Fucα1-4)GlcNAcβ-sp3	3'-O-Su-Le^{a}	532	0	942	1
288	3-O-Su-Galβ1-4(Fucα1-3)GlcNAcβ-sp3	3'-O-Su-Le^{x}	2314	1	283	0
289	Neu5Acα2-6(Galβ1-3)GalNAcα-sp3		746	0	1834	2
290	Neu5Acα2-6(Galα1-3)GalNAcα-sp3		410	0	1350	1
291	Neu5Acβ2-6(Galβ1-3)GalNAcα-sp3		3034	1	4043	4
292	Neu5Acα2-3Galβ1-3GalNAcα-sp3		559	0	1716	1
293	Neu5Acα2-3Galβ1-4Glcβ-sp3	3'SL	516	0	777	0
294	Neu5Acα2-3Galβ1-4Glcβ-sp4	3'SL	9946	4	1779	2
295	Neu5Acα2-6Galβ1-4Glcβ-sp2	6'SL	442	0	691	1
296	Neu5Acα2-6Galβ1-4Glcβ-sp4	6'SL	1172	1	1959	2
297	Neu5Acβ2-6Galβ1-4Glcβ-sp2		703	0	3047	2
298	Neu5Acα2-3Galβ1-4GlcNAcβ-sp3	3'SLN	286	0	409	0
299	Neu5Acα2-3Galβ1-3GlcNAcβ-sp3	3'SiaLeC	3900	5	10595	7
300	Neu5Acα2-6Galβ1-4GlcNAcβ-sp3	6'SLN	333	0	399	0
302	Neu5Acβ2-6Galβ1-4GlcNAcβ-sp3		745	0	2542	2
303	Neu5Gcα2-3Galβ1-4GlcNAcβ-sp3		296	0	475	0
304	Neu5Gcα2-6Galβ1-4GlcNAcβ-sp3		345	0	323	0
305	Neu5Gcβ2-6Galβ1-4GlcNAcβ-sp3		829	0	1399	1
306	9-NAc-Neu5Acα2-6Galβ1-4GlcNAcβ-sp3		498	0	4101	2
307	KDNα2-3Galβ1-3GlcNAcβ-sp2		2710	1	10405	7
308	KDNα2-3Galβ1-4GlcNAcβ-sp2		571	0	2363	2
309	Neu5Acα2-6(Neu5Acα2-3)GalNAcα-sp3		307	0	527	0
310	Neu5Acα2-3Galβ1-4GlcNAcβ-$O(CH_2)_3$NH-amide-Neu5Acα2-3Galβ1-4GlcNAcβ-sp3		325	0	262	0
315	Neu5Acα2-3Galβ1-4-(6-O-Su)GlcNAcβ-sp3		362	0	460	0
316	Fucα1-2(6-O-Su)Galβ1-4GlcNAcβ-sp3	6'-suH(type2)	365	0	420	0
318	Neu5Acα2-6Galβ1-4(6-O-Su)GlcNAcβ-sp3		341	0	624	0
319	Neu5Acα2-3(6-O-Su)Galβ1-4GlcNAcβ-sp3		276	0	306	0
320	4-O-Su-Neu5Acα2-3(6-O-Su)Galβ1-4GlcNAcβ-sp3		417	0	811	0
321	(Neu5Acα2-8)$_3$-sp3	(Sia)3	300	0	350	0
322	(Neu5Acα2-8)$_3$β-sp3		625	0	3557	3
323	Neu5Acα2-6Galβ1-3GlcNAcβ-sp3	6'-SiaLe^{c}	916	0	2338	3

324	Neu5Acα2-6Galβ1-3(6-O-Su)GlcNAcβ-sp3		909	0	1249	1
325	Neu5Acα2-3Galβ1-4Glcβ-sp4-Ala		10125	6	1355	1
326	Neu5Acα2-3Galβ1-4Glcβ-sp4-Ile		10081	6	1340	1
327	Neu5Acα2-3Galβ1-4Glcβ-sp4-Nle		11318	6	1616	1
328	Neu5Acα2-3Galβ1-4Glcβ-sp4-Phe		10120	5	1378	1
329	Neu5Acα2-3Galβ1-4Glcβ-sp4-Trp		13038	6	2253	2
330	Neu5Acα2-3Galβ1-4Glcβ-sp4-Val		11273	6	1070	0
331	Neu5Gcα2-3Galβ1-3GlcNAcβ-sp3		4086	5	11438	7
332	Neu5Acα2-3Galβ1-4Glcβ-sp4-Asn		8253	6	1138	0
333	Neu5Gcα2-3Galβ1-3(6-O-Su)GlcNAcβ-sp3		1627	1	2619	2
334	Neu5Gcα2-3Galβ1-4(6-O-Su)GlcNAcβ-sp3		1083	1	691	0
335	Neu5Acα2-3Galβ1-3(6-O-Su)GlcNAcβ-sp3		1467	1	1703	2
336	αKdo-(2→8)-αKdo-(2→4)-αKdo-sp11		8196	6	8844	6
337	GalNAcα1-4Galβ1-4GlcNAcβ-sp3		3479	5	7396	7
338	Neu5Acα2-6Galβ1-3GalNAcα-sp3	6'-sialyl-TF	1124	1	1195	0
339	Neu5Acβ2-6Galβ1-3GalNAcα-sp3		1024	0	5501	5
340	Galα1-3(Neu5Acβ2-6)GalNAcβ-sp3		935	0	5384	5
341	Neu5Acα2-3-(6-O-Su)Galβ1-4GlcNAcβ-sp2		287	0	388	0
359	Galα1-3(Fucα1-2)Galβ1-3GlcNAcβ-sp3	B (type 1)	5790	3	4958	3
360	Galα1-3(Fucα1-2)Galβ1-4GlcNAcβ-sp3	B (type 2)	976	1	2409	3
361	Galα1-3(Fucα1-2)Galβ1-4GlcNAcβ-sp2	B (type 2)	879	0	2360	3
362	Galα1-3(Fucα1-2)Galβ1-3GalNAcα-sp3	B (type 3)	2839	2	3936	4
363	Galα1-3(Fucα1-2)Galβ1-3GalNAcβ-sp3	B (type 4)	4122	2	3028	3
364	Galα1-3Galβ1-4(Fucα1-3)GlcNAcβ-sp3	αGalLe^{x}	1844	2	1224	0
365	Galα1-4(Fucα1-2)Galβ1-4GlcNAcβ-sp3		1364	0	2913	3
366	GalNAcα1-3(Fucα1-2)Galβ1-3GlcNAcβ-sp3	A (type 1)	6857	5	5894	6
368	GalNAcα1-3(Fucα1-2)Galβ1-4GlcNAcβ-sp3	A (type 2)	2223	1	4757	4
369	GalNAcα1-4(Fucα1-2)Galβ1-4GlcNAcβ-sp3		2407	2	10296	5
370	GalNAcβ1-3(Fucα1-2)Galβ1-4GlcNAcβ-sp3		500	0	1364	1
371	Fucα1-2Galβ1-3(Fucα1-4)GlcNAcβ-sp3	Le^{b}	984	0	2419	2
372	Fucα1-2Galβ1-4(Fucα1-3)GlcNAcβ-sp3	Le^{y}	731	1	307	0
373	Galα1-3Galβ1-4GlcNAcβ1-3Galβ-sp3	Galili (tetra)	4446	2	5824	4
374	Galα1-3(Galα1-4)Galβ1-4GlcNAcβ-sp3		1653	1	3479	5
375	Galα1-4GlcNAcβ1-3Galβ1-4GlcNAcβ-		9635	8	10750	8

	sp3					
376	Galβ1-3GlcNAcβ1-3Galβ1-4Glcβ-sp4	N-acetyllacto-N-tetraose, LNT	15939	6	3004	4
377	Galβ1-3GlcNAcβ1-3Galβ1-3GlcNAcβ-sp2		2338	2	8026	6
378	Galβ1-3GlcNAcα1-3Galβ1-4GlcNAcβ-sp3		17225	9	12928	8
379	Galβ1-3GlcNAcβ1-3Galβ1-4GlcNAcβ-sp3		512	0	913	1
380	Galβ1-3GlcNAcα1-6Galβ1-4GlcNAcβ-sp2		17866	9	6284	6
381	Galβ1-3GlcNAcβ1-6Galβ1-4GlcNAcβ-sp2		809	0	1027	1
382	Galβ1-3GalNAcβ1-4Galβ1-4Glcβ-sp3	GA1, asialo-GM1	2614	2	7904	7
383	Galβ1-4GlcNAcβ1-3Galβ1-4Glcβ-sp4	N-acetyllacto-neo-tetraose, LNnT	10513	6	2315	3
384	Galβ1-4GlcNAcβ1-3Galβ1-4GlcNAcβ-sp2	i	777	1	324	0
386	Galβ1-4GlcNAcα1-6Galβ1-4GlcNAcβ-sp2		4895	3	7764	5
387	Galβ1-4GlcNAcβ1-6Galβ1-4GlcNAcβ-sp2		2520	1	1290	1
388	Galβ1-4GlcNAcβ1-6(Galβ1-3)GalNAcα-sp3		986	0	1576	2
389	GalNAcβ1-3Galα1-4Galβ1-4Glcβ-sp3	Gb4, P	7335	5	7361	5
390	(Glcα1-4)$_4$β-sp4	maltotetraose	1660	0	4170	2
392	GalNAcα1-3(Fucα1-2)Galβ1-3GalNAcα-sp3	A (type 3)	7921	4	8230	6
394	GlcNAcβ1-4(GlcNAcβ1-3)Galβ1-4GlcNAcβ-sp2		3171	4	4225	3
395	GlcNAcβ1-6(GlcNAcβ1-3)Galβ1-4GlcNAcβ-sp2	T_k	6193	1	861	1
396	(GlcNAcβ1)$_3$-3,4,6-GalNAcα-sp3		4518	4	9667	7
397	Galβ1-3GlcN(Fm)β1-3Galβ1-4GlcNAcβ-sp3		11759	8	13243	7
398	Galβ1-3GlcN(Fm)β1-3Galβ1-3GlcNAcβ-sp3		4683	5	13263	8
399	Galβ1-3GlcNAcα1-3Galβ1-3GlcNAcβ-sp2		10316	9	14915	9
401	Galβ1-3GlcNAcβ1-3Galβ1-3GlcNAcβ-sp3		2680	3	8965	6
404	GalNAcα1-3Galβ1-4(Fucα1-3)GlcNAcβ-sp3	αGalNAcLe$_x$	3138	1	7775	6
405	Galα1-3(Fucα1-2)Galα1-4GlcNAcβ-sp3		1374	1	2046	2
406	GalNAcα1-3(Fucα1-2)Galα1-3GalNAcβ-sp3		4198	2	4605	5

419	3-O-SuGalβ1-4GlcNAcβ1-3Galβ1-4GlcNAcβ-sp3		464	0	499	0
420	4-O-SuGalβ1-4GlcNAcβ1-3Galβ1-4GlcNAcβ-sp3		4601	4	8092	7
423	Neu5Acα2-3Galβ1-4(Fucα1-3)GlcNAcβ-sp3	$SiaLe^x$	262	0	241	0
425	Neu5Acα2-3Galβ1-4(Fucβ1-3)GlcNAcβ-sp3		1689	1	317	0
426	Neu5Acα2-3Galβ1-3(Fucα1-4)GlcNAcβ-sp3	$SiaLe^a$	311	0	236	0
428	Neu5Acα2-3Galβ1-4(Fucα1-3)(6-O-Su)GlcNAcβ-sp3	6-O-Su-$SiaLe^x$	505	0	253	0
429	Neu5Acα2-3(6-O-Su)Galβ1-4(Fucα1-3)GlcNAcβ-sp3	6'-O-Su-$SiaLe^x$	292	0	369	0
431	Neu5Acα2-3Galβ1-4(2-O-Su-Fucα1-3)GlcNAcβ-sp3		238	0	244	0
432	Neu5Acα2-3Galβ1-4(3-O-Su-Fucα1-3)GlcNAcβ-sp3		462	0	395	0
433	Neu5Acα2-6(Neu5Acα2-3Galβ1-3)GalNAcα-sp3		834	1	178	0
434	Neu5Acα2-8Neu5Acα2-3Galβl-4Glcβ-sp4	GD3	6320	4	1221	1
435	Neu5Acα2-3Galβ1-4(2-O-Su-Fucα1-3)(6-O-Su)GlcNAcβ-sp3		374	0	573	1
436	4-O-Su-Neu5Acα2-3Galβ1-4(Fucα1-3)(6-O-Su)GlcNAcβ-sp3		472	0	903	0
437	GalNAcα1-3(Fucα1-2)Galβ1-3GalNAcβ-sp3	A(type 4)	10036	5	6650	5
438	Fucβ1-2Galβ1-4(Fucα1-3)GlcNAcβ-sp3		805	0	2364	3
439	Kdoα2-4Kdoα2-4Kdoα2-6GlcNAcβ-sp11		4774	3	6827	5
440	Neu5Acβ2-6(Fucα1-2)Galβ1-4GlcNAcβ-sp3		748	0	3748	4
441	Neu5Acα2-6(Fucα1-2)Galβ1-4GlcNAcβ-sp3		245	0	147	0
479	Fucα1-2Galβ1-3GlcNAcβ1-3Galβ1-4Glcβ-sp4	H(type1), LNFP I	7304	4	1949	2
480	Fucα1-2Galβ1-3GlcNAcβ1-3Galβ1-4GlcNAcβ-sp2	H(type 1) penta	316	0	370	0
481	Galα1-3Galβ1-4GlcNAcβ1-3Galβ1-4Glcβ-sp4	Galili (penta)	11987	6	6156	5
482	Galα1-3(Fucα1-2)Galβ1-3(Fucα1-4)GlcNAcβ-sp3	BLe^b	1494	1	1311	1
483	Galα1-3(Fucα1-2)Galβ1-4(Fucα1-3)GlcNAcβ-sp3	BLe^y	1006	1	1016	0
484	GalNAcα1-3(Fucα1-2)Galβ1-3(Fucα1-4)GlcNAcβ-sp3	ALe^b	1548	1	1971	2
485	Galβ1-4GalNAcα1-3(Fucα1-2)Galβ1-4GlcNAcβ-sp3		1733	1	3789	4
491	GalNAcα1-3(Fucα1-2)Galβ1-4(Fucα1-3)GlcNAcβ-sp3	ALe^y	1804	2	3697	4
492	$(Glcα1\text{-}6)_5β$-sp4	isomaltopentaose	1746	1	1824	1
493	$(GlcNAcβ1\text{-}4)_5β$-sp4	chitopentaose	2162	1	3545	5

495	Manα1-6(Manα1-3)Manα1-6(Manα1-3)Manβ-sp4	Man_5	507	0	952	1
496	Fucα1-2Galβ1-3(Fuca1-4)GlcNAcβ1-3Galβ1-4Glcβ-sp4	Le^b(hexa)	7203	3	3248	3
497	Fucα1-2Galβ1-4(Fucα1-3)GlcNAcβ1-3Galβ1-4Glcβ-sp4	Le^y (hexa)	6252	3	2006	2
498	Galβ1-4GlcNAcβ1-3Galβ1-4GlcNAcβ1-3Galβ1-4GlcNAcβ-sp3	$(LN)_3$	323	0	375	0
499	Galβ1-4GlcNAcβ1-6(Galβ1-4GlcNAcβ1-3)Galβ1-4GlcNAcβ-sp2	I	671	1	203	0
501	Galβ1-3GalNAcβ1-3Galα1-4Galβ1-4Glcβ-sp4	Gb5	6516	5	9659	8
502	$(Glc\alpha1\text{-}6)_6\beta$-sp4	isomaltohexaose	1947	2	1614	1
503	$(GlcNAc\beta1\text{-}4)_6$-sp4	chitohexaose	2022	2	3749	4
504	$(A\beta1\text{-}4GN\beta1\text{-}2M\alpha1)_2$-3,6-Mβ1-4GNβ1-4GNβ-sp4		267	0	142	0
505	$(GN\beta1\text{-}2M\alpha1)_2$-3,6-Mβ1-4GNβ1-4GNβ-sp4		260	0	183	0
506	Ara*f*β1-2Ara*f*α1-5(Ara*f*β1-2Ara*f*α1-3)Ara*f*α1-5Ara*f*α-$O(CH_2)_2NHCOCH_2(OCH_2CH_2)_6NH_2$	Ara_6	10893	6	9096	6
507	GalNAcα1-3GalNAcβ1-3Galα1-4Galβ1-4Glcβ-sp3	Fs-5	4148	5	16634	10
508	GalNAcβ1-3(Fucα1-2)Galβ1-4(Fucα1-3)GlcNAcβ-sp3		764	0	1626	1
509	Galβ1-3GalNAcβ1-4(Neu5Acα2-3)Galβ1-4Glcβ-sp4	GM1	513	0	1247	1
527	Neu5Acα2-3Galβ1-4GlcNAcβ1-3Galβ1-4GlcNAcβ-sp2		388	0	391	0
528	Neu5Acα2-3Galβ1-4(Fucα1-3)GlcNAcβ1-3Galβ-sp3	$SiaLe^x$-Gal	409	0	508	0
529	Neu5Acα2-6(Galβ1-3)GlcNAcβ1-3Galβ1-4Glcβ-sp4	LSTb	4160	3	1777	1
530	$(Neu5Ac\alpha2\text{-}3Gal\beta1)_2$-3,4-GlcNAcβ-sp3		218	0	152	0
531	Neu5Acα2-8Neu5Acα2-3(GalNAcβ1-4)Galβ1-4Glcβ-sp2	GD2	641	0	2069	1
534	Neu5Acα2-6Galβ1-4GlcNAcβ1-3Galβ1-4GlcNAcβ-sp3		1161	1	290	0
535	Neu5Acα2-8Neu5Acα2-3(GalNAcβ1-4)Galβ1-4Glcβ-sp4	GD2	1524	2	850	1
536	Neu5Aα2-3Galβ1-3GlcNAcβ1-3Galβ1-4Glcβ-sp4	LSTa	10168	5	2332	3
537	Neu5Aα2-3Galβ1-4GlcNAcβ1-3Galβ1-4Glcβ-sp4	LSTd	9594	5	2335	3
538	Galβ1-4(Fucα1-3)GlcNAcβ1-6(Galβ1-3GlcNAcβ1-3)Galβ1-4Glcβ-sp4	MFLNH III	2235	2	782	1
539	Galβ1-4GlcNAcβ1-6(Fucα1-2Galβ1-3GlcNAcβ1-3)Galβ1-4Glcβ-sp4	MFLNH I	415	0	224	0
540	Galβ1-4(Fucα1-3)GlcNAcβ1-6(Neu5Acα2-6Galβ1-4GlcNAcβ1-3)Galβ1-4Glcβ-sp4	MSMFLNnH	938	1	552	1

541	Galβ1-4(Fucα1-3)GlcNAcβ1-6(Fucα1-2Galβ1-3GlcNAcβ1-3)Galβ1-4Glcβ-sp4	DFLNH (a)	1236	1	769	1
542	Galβ1-3GlcNAcβ1-3Galβ1-4(Fucα1-3)GlcNAcβ1-6(Galβ1-3GlcNAcβ1-3)Galβ1-4Glcβ-sp4	MF(1-3)iLNO	660	0	1937	2
545	Kdoα2-8Kdoα2-4Kdoα2GlcNAcβ1-6GlcNAcα-sp11		8516	6	8088	8
625	(GlcAβ1-3GlcNAcβ1-4)$_{11-12}$-NH$_2$-ol	hyaluronic acid	634	0	3389	1
627	(Siaα2-6Aβ1-4GNβ1-2Mα1)2-3,6-Mβ1-4GNβ1-4GNβ-sp4	11-OS, YDS	269	0	136	0
629	Trehalose-ethanolamine		344	0	995	1
630	(GlcAβ1-3GlcNAcβ1-4)$_{20}$-NH(*p*-C$_6$H$_4$)CH$_2$CH$_2$NH$_2$	hyaluronic acid	2342	2	1728	2
631	(GlcAβ1-3GlcNAcβ1-4)$_{38}$-NH(*p*-C$_6$H$_4$)CH$_2$CH$_2$NH$_2$	hyaluronic acid	755	1	676	0
632	(GlcAβ1-3GlcNAcβ1-4)$_{13}$-NH(*p*-C$_6$H$_4$)CH$_2$CH$_2$NH$_2$	hyaluronic acid	710	0	2377	1
800	GlcNAcα1-4GlcNAcβ-sp3		8580	5	9035	6
801	GalNAcα1-3GalNAc(fur)β-sp3		2985	3	6799	5
802	Galβ1-3GalNAc(fur)β-sp3		2236	1	9471	8
804	[Galβ1-4GlcNAcβ-OCH$_2$CH$_2$]$_2$NH		360	0	580	0
805	GalNAcβ1-4(6-O-Bn)GlcNAcβ-sp3		1490	1	5025	4
806	Galα1-6Glcα-sp3		14739	9	6689	8
807	GlcNAcβ1-4GlcNAcα-sp4		7505	8	7435	7
808	Galα1-6Glcβ-sp3	melibiose	15978	10	6956	8
809	GalNAcβ1-3GalNAcα-sp3		4753	4	6160	7
810	GalNGcα1-3GalNAcα-sp3		1106	0	8676	4
850	Galβ1-3(6-O-Su)GalNAcα-sp3	6-suTF	991	0	4145	4
851	Galα1-3(6-O-Su)GalNAcα-sp3		723	0	2569	3
852	GlcNAcβ1-4-[HOOC(CH$_3$)CH]-3-O-GlcNAcα-sp4		6242	3	4057	5

sp2 = -O(CH$_2$)$_2$NH$_2$

sp3 = -O(CH$_2$)$_3$NH$_2$

sp4 = -NHCOCH$_2$NH$_2$

sp5 = -O(CH$_2$)$_3$NH-CO(CH$_2$)$_5$NH$_2$

sp8 = -(OCH$_2$CH$_2$)$_6$NH$_2$
